# Edge‐hosted Atomic Co−N_4_ Sites on Hierarchical Porous Carbon for Highly Selective Two‐electron Oxygen Reduction Reaction

**DOI:** 10.1002/anie.202213296

**Published:** 2022-11-17

**Authors:** Yuhui Tian, Meng Li, Zhenzhen Wu, Qiang Sun, Ding Yuan, Bernt Johannessen, Li Xu, Yun Wang, Yuhai Dou, Huijun Zhao, Shanqing Zhang

**Affiliations:** ^1^ Centre for Catalysis and Clean Energy School of Environment and Science Gold Coast Campus Griffith University Queensland 4222 Australia; ^2^ Centre for Microscopy and Microanalysis University of Queensland Brisbane Queensland, 4072 Australia; ^3^ Institute of Energy Materials Science University of Shanghai for Science and Technology Shanghai 200093 China; ^4^ Australia Synchrotron Australia's Nuclear Science and Technology Organization Victoria 3168 Australia; ^5^ Institute for Energy Research School of Chemistry and Chemical Engineering Key Laboratory of Zhenjiang Jiangsu University Zhenjiang 212013 China; ^6^ Shandong Institute of Advanced Technology Jinan 250103 China

**Keywords:** H_2_O_2_ Production, Oxygen Reduction Reaction, Single-Atom Catalysts

## Abstract

Not only high efficiency but also high selectivity of the electrocatalysts is crucial for high‐performance, low‐cost, and sustainable energy storage applications. Herein, we systematically investigate the edge effect of carbon‐supported single‐atom catalysts (SACs) on oxygen reduction reaction (ORR) pathways (two‐electron (2 e^−^) or four‐electron (4 e^−^)) and conclude that the 2 e^−^‐ORR proceeding over the edge‐hosted atomic Co−N_4_ sites is more favorable than the basal‐plane‐hosted ones. As such, we have successfully synthesized and tuned Co‐SACs with different edge‐to‐bulk ratios. The as‐prepared edge‐rich Co−N/HPC catalyst exhibits excellent 2 e^−^‐ORR performance with a remarkable selectivity of ≈95 % in a wide potential range. Furthermore, we also find that oxygen functional groups could saturate the graphitic carbon edges under the ORR operation and further promote electrocatalytic performance. These findings on the structure–property relationship in SACs offer a promising direction for large‐scale and low‐cost electrochemical H_2_O_2_ production via the 2 e^−^‐ORR.

## Graphical abstract

The edge‐hosted Co−N_4_ sites in the carbon matrix are more efficient and selective than basal plane‐hosted ones in catalyzing 2 e^−^‐ORR, achieving a remarkable selectivity of ≈95 % in the electrochemical production of H_2_O_2_.

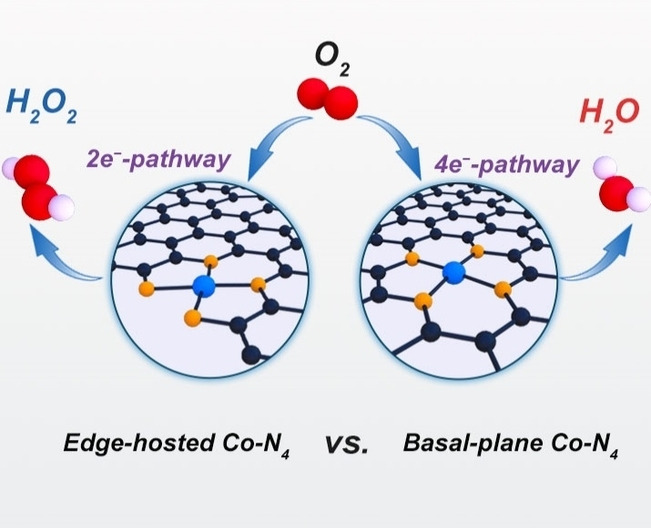

## Introduction

Sustainable and clean sources for producing critical chemicals are highly desirable for realizing the goal of carbon neutrality or “net‐zero” emission in modern societies. As a value‐added chemical, hydrogen peroxide (H_2_O_2_) is an eco‐friendly oxidant widely used in the household, medical, agricultural, and industrial sectors.^[1]^ To date, the industrial manufacturing of H_2_O_2_ is almost exclusively through the anthraquinone method, an energy‐consuming process requiring costly noble‐metal Pd catalysts.^[1]^ Moreover, the consumption and generation of organic compounds in this process could lead to substantial environmental hazards.^[2]^

Much research in recent years has beckoned the exploitation of the electrocatalytic oxygen reduction reaction (ORR) to produce H_2_O_2_ through a more “green” and sustainable scheme, in which the selective two‐electron (2 e^−^) reduction of O_2_ at the cathode is of vital importance towards low‐cost and efficient H_2_O_2_ production.^[3]^ This approach enables the integration of renewable electricity as the energy supply for sustainable production of H_2_O_2_ from earth‐abundant raw materials (air and water), with no hazardous by‐products produced. However, the major challenge of the electrosynthesis of H_2_O_2_ is that the ORR process would occur through the competitive four‐electron (4 e^−^) pathway, which substantially reduces the production yield.^[4]^ Therefore, besides the activity and stability of the electrocatalyst materials, a high level of selectivity for the targeted reaction route is becoming the most important key to tackling this challenge.

Single‐atom catalysts (SACs) with isolated metal atoms dispersed on substrates are rising stars in heterogeneous catalysis.^[5]^ In SACs, the catalytic activity is governed by the local environment of atomic metal moieties, which can be finely tuned by replacing the metal center and adjusting surrounding atoms. Such a flexible tunability of SACs provides limitless opportunities to simultaneously achieve high selectivity and productivity for the electrochemical synthesis of H_2_O_2_. Moreover, the lack of contiguous metal ensemble sites in SACs would prevent the decomposition or further electrochemical reduction of generated H_2_O_2_.^[6]^ Recently, carbon‐supported Co‐SACs with the Co−N−C coordination configuration have shown desirable 2 e^−^‐ORR performances with a selectivity >80 %.^[7]^ In strong contrast, Co‐SACs have also been reported as state‐of‐the‐art electrocatalysts for the 4 e^−^‐ORR.^[8]^ Therefore, thorough identification of catalyst structures and a comprehensive understanding of the reaction mechanism are urgently needed to realize selective 2 e^−^‐ or 4 e^−^‐ORR for the targeted electrochemical applications.

It is well‐established that the atomically dispersed metal moieties exhibit a variety of anchoring positions and local coordination environments across the carbon substrate. For example, the graphitic carbon edge is a particular anchoring point for favorable immobilization of atomic metal species.^[9]^ Compared to the basal‐plane sites, the dangling bonds located at the edge of *sp* ^*2*^ hybridized C atoms can induce the redistribution of local electrons and lead to altered bonding environments adjacent to atomic metal moieties.^[10]^ Consequently, basal‐plane‐ and edge‐hosted atomic metal sites are markedly different in their intrinsic catalytic properties.^[11]^ However, previously investigated Co‐SACs are mainly simplified by the Co−N_4_ site embedded in the basal plane of bulk graphene. The activation mainly involves tailoring the local coordination environment of the Co site (i.e., the first and second coordination shells).^[12]^ The local geometries of trapping states at defective pockets and edges of the carbon matrix and corresponding properties are quite neglected. Moreover, chemical changes on the carbon surface under the electrochemical operation and corresponding influences on ORR performances have not yet been well investigated.

Aimed to close these fundamentally important knowledge gaps, we propose to manipulate the ORR pathway on Co‐SACs via the edge effect of the carbon matrix. Our density functional theory (DFT) calculations suggest that edge‐hosted atomic Co−N_4_ sites are more thermodynamically favorable for 2 e^−^‐ORR than the basal‐plane sites. Experimentally, atomically dispersed Co−N_4_ moieties are immobilized on carbon substrates with different edge‐to‐bulk ratios. When Co−N_4_ sites are anchored on hierarchically porous carbon (HPC), the resultant Co−N/HPC catalyst with rich edge‐hosted active sites presents a high selectivity of ≈95 % for 2 e^−^‐ORR in alkaline media. In contrast, basal‐plane Co−N_4_ dominated graphene flakes (Co−N/GFs) demonstrate a quasi‐4 e^−^ pathway towards the ORR. More importantly, we find that oxygen‐containing functional groups (OFGs) could readily saturate the edges of the carbon host during the long‐term ORR operation. Further experimental and theoretical investigations disclose that the modification of OFGs can further promote the 2 e^−^‐ORR kinetics while maintaining the high selectivity above 90 %. This study provides mechanistic insights into the origin of the high selectivity of 2 e^−^‐ORR at Co‐SACs.

## Results and Discussion

**Figure 1:**
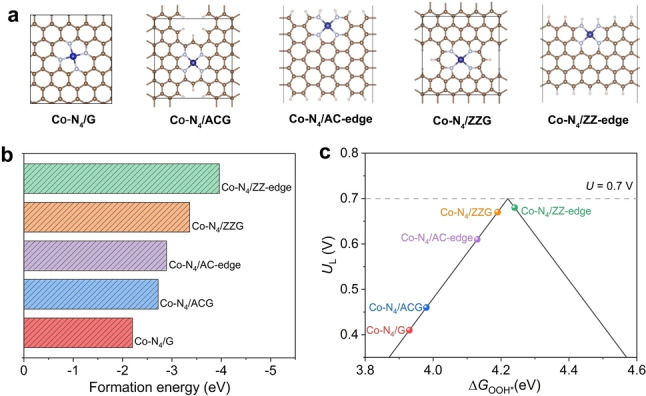
a) The optimized structural models of the basal‐plane Co−N_4_/G and edge‐hosted Co−N_4_ structures, including Co−N_4_/ACG, Co−N_4_/AC‐edge, Co−N_4_/ZZG, and Co−N_4_/ZZ‐edge. Color code: the brown, grey, blue, and pink balls refer to C, N, Co, and H atoms, respectively. b) Calculated formation energies of different Co−N−C structures. c) The calculated volcano plot for 2 e^−^‐ORR. The *U*_L_ is plotted as a function of Δ*G*_OOH*_.

DFT calculations were firstly conducted to theoretically study the correlations between the reaction energetics of ORR and the local geometry of anchoring sites. Five types of Co−N_4_ configurations were constructed based on the anchoring positions over defective sites of monolayer graphene (Figure 1a), including basal‐plane‐hosted Co−N_4_/G (located in the graphene basal plane), edge‐hosted Co−N_4_/ACG (located between graphene armchair edges), Co−N_4_/AC‐edge (situated beside the graphene armchair edge), Co−N_4_/ZZG (located between graphene zigzag edges), and Co−N_4_/ZZ‐edge (situated beside the graphene zigzag edge).^[13]^ The thermodynamic stability of SACs strongly depends on the anchoring sites in host materials and their interactions with the coordination‐unsaturated metal atoms.^[14]^ Thus, the formation energies of the constructed Co−N_4_ configurations were first compared by calculating the energy changes when the Co atom was embedded into the N‐doped graphene matrix.^[15]^ As shown in Figure 1b, the calculated formation energies of Co−N_4_/ACG, Co−N_4_/AC‐edge, Co−N_4_/ZZG, and Co−N_4_/ZZ‐edge are lower than that of Co−N_4_/G, suggesting that Co atoms prefer to be confined over the edge sites by forming the Co−N bond.^[15]^

The Gibbs free energies for ORR on these structures were then calculated (Figure S1–2, Supporting Information). For 2 e^−^‐ORR, the catalytic activity is determined by either the step of O_2_ activation or OOH* desorption. Therefore, the adsorption‐free energy of OOH* (Δ*G*_OOH*_) can be used as the key descriptor to predict the reaction activity.^[16]^ Ideally, the Δ*G*_OOH*_ for the 2 e^−^‐ORR should be ≈4.2 eV, and thus the thermodynamic limiting potential (*U*_L_) is close to the equilibrium potential of 0.7 V with minimized overpotential.^[12c]^ For the 4 e^−^‐ORR process, the optimal Δ*G*_OOH*_ should be close to 3.9 eV.^[12b]^ Based on the calculated results, the activity volcano plot (Figure 1c) was constructed as a function of Δ*G*_OOH*_. Co−N_4_/G is at the left leg of the volcano with Δ*G*_OOH*_ of 3.93 eV. Such a strong OOH* binding tends to drive the ORR towards the 4 e^−^‐pathway.[^12b^, ^12c^] In contrast, the binding strength of OOH* is weakened when Co−N_4_ resides at the defective pockets and edge sites. Remarkably, Co−N_4_/ZZ‐edge is near the peak of the volcano plot with a moderate Δ*G*_OOH*_ of 4.24 eV and almost zero overpotential for H_2_O_2_ production. For the 4 e^−^‐pathway, the formation of OOH* is the rate‐determining step for Co−N_4_/ACG, Co−N_4_/AC‐edge, Co−N_4_/ZZG, and Co−N_4_/ZZ‐edge with higher energy barriers than that of Co−N_4_/G (Figure S3), indicating their less desirable 4 e^−^‐ORR activity. These results suggest that the location of anchoring sites has a significant influence on the ORR energetics, and the edge‐hosted Co−N_4_ sites are more favorable for 2 e^−^‐ORR than that over the basal‐plane‐hosted site.

The edge effect on Δ*G*_OOH*_ can be explained by the differences in electronic properties of the central Co atom over different configurations. As examined by charge differences of Co centers (Figure S4), noticeable differences in the charge distributions are observed for the five atomic Co−N_4_ models. The Bader charge analyses show that the charge value of the Co atom is estimated to be +0.87 |e| for Co−N_4_/G while becoming more positive for other models (Table S1). The increased positive charge state indicates that the Co atoms at the defective pockets and edge sites carry less charge than at the basal plane.^[17]^ Meanwhile, the partial density of states (PDOS) of Co 3d‐orbitals shows lower *d*‐band center positions than Co−N_4_/G (Figure S5), thus leading to weakened binding with the intermediates.^[18]^ Therefore, the break of plane symmetry caused by edges can considerably modulate the electronic structure of the Co atom by altering the charge state and downshifting the *d*‐band center. According to our theoretical results, it can be expected that a highly efficient Co‐SAC for electrosynthesis of H_2_O_2_ via 2 e^−^‐ORR can be practically obtained by enriching the edge‐hosted Co−N_4_ sites or using edge‐abundant carbon substrates.

**Figure 2:**
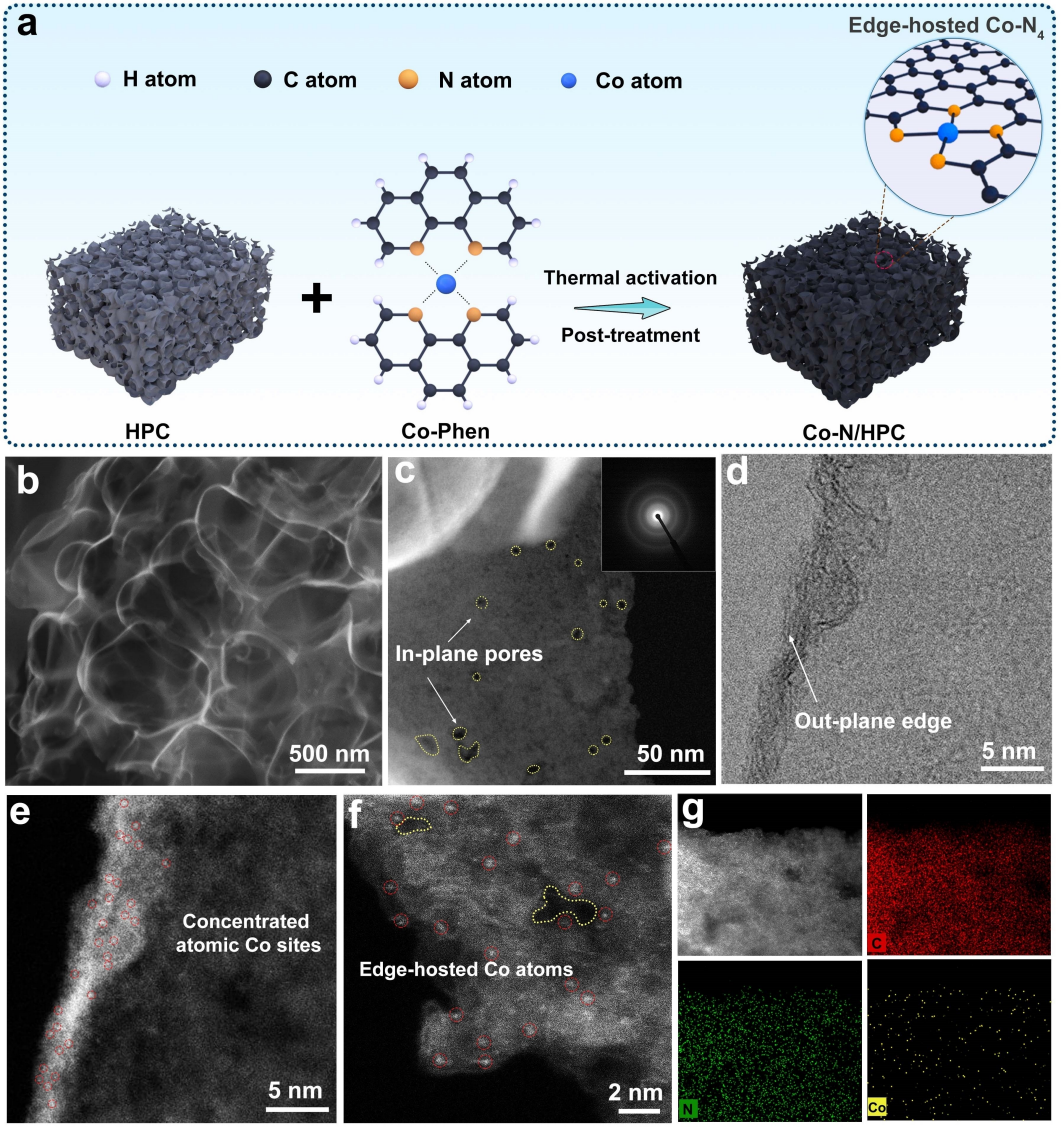
a) Schematic illustration of the fabrication procedure of Co−N/HPC catalyst from hierarchically porous carbon (HPC) and Co‐Phen. b) SEM image, c) DF‐TEM image (inset: SAED pattern), d) BF‐STEM image, and e–f) HAADF‐STEM images of Co−N/HPC. g) HAADF‐STEM and corresponding elemental mapping images of Co−N/HPC.

Enlightened by the theoretical investigation, atomic dispersion of Co on the carbon substrate was achieved via a simple ligand‐assisted impregnation‐calcination method. The fabrication process is briefly illustrated in Figure 2a. Typically, 1,10‐phenanthroline (Phen) was selected as the ligand to coordinate Co^2+^ ions to form the Co‐Phen complex. The hierarchically porous carbon (HPC) and graphene flakes (GFs) with various edge‐to‐bulk ratios were utilized as the host materials to adsorb the Co‐Phen complex via wet impregnation. After thermal activation and further post‐treatment to remove unstable species (see details in the Experimental Section in Supporting Information), Co−N/HPC and Co−N/GFs catalysts were obtained.

X‐ray diffraction (XRD) patterns (Figure S6) show that the diffraction peak of the graphitic carbon (002) at ≈25.8° for Co−N/HPC is much broader than Co−N/GFs, indicating a low degree of graphitization and a defect‐rich feature.^[12a]^ No characteristic diffraction peaks of crystalline Co species are detected in both samples, suggesting the possible formation of atomically dispersed Co moieties. The scanning electron microscopy (SEM) image shows the hierarchically porous framework of Co−N/HPC with rich edges (Figure 2b). As a sharp contrast, the Co−N/GFs catalyst displays crumpled sheet‐like morphology with much fewer exposed edges (Figure S7). A close‐up of the dark‐field transmission electron microscopy (DF‐TEM) image of Co−N/HPC (Figure 2c) shows abundant irregular micropores and mesopores on the basal plane, which can provide additional in‐plane pore edges as anchoring sites for Co atoms. Additionally, the flourishing porosity facilitates the rapid diffusion of generated H_2_O_2_ into the bulk electrolyte.^[19]^ The selected area electron diffraction (SAED) pattern of Co−N/HPC exhibits the typical feature of amorphous carbon with blurry diffraction rings (inset of Figure 2c), further excluding the existence of crystalline Co species.^[20]^

The aberration‐corrected scanning transmission electron microscopy (STEM) was employed to examine the dispersion of Co species at the atomic level. The bright‐field (BF) image shows that Co−N/HPC exhibits abundant ripples (Figure 2d), which are recognized as out‐of‐plane edges.^[21]^ The high‐angle annular dark‐field (HAADF) image (Figure 2e) of the same area displays concentrated atomic‐scale bright dots (marked by the red dashed cycles), which are identified as the isolated Co single atoms with a diameter of ≈0.19 nm.^[12a]^ Moreover, single‐atomic Co dopants can also be found around the in‐plane pore edges (Figure 2f). In contrast, single‐atomic Co species are mainly dispersed in the basal plane of Co−N/GFs due to limited edge structures (Figure S8). The ratio of isolated Co atoms is over 96 % for Co−N/HPC and Co−N/GFs (Figure S9), indicating the significant amount of isolated Co species in prepared catalysts. The edge‐hosted Co atoms in Co−N/HPC are estimated to be 20.4 % via statistical analysis (Figure S10), which is over three times higher than Co−N/GFs (6.3 %), suggesting the critical role of pore‐ and edge‐rich HPC in exposing edge‐hosted Co species. Energy‐dispersive X‐ray spectroscopy (EDS) mapping of Co−N/HPC shows the existence of N and Co throughout the carbon matrix (Figure 2g). Inductively coupled plasma mass spectrometry revealed that the Co loadings were 0.21 and 0.22 wt % for Co−N/HPC and Co−N/GFs, respectively.

**Figure 3:**
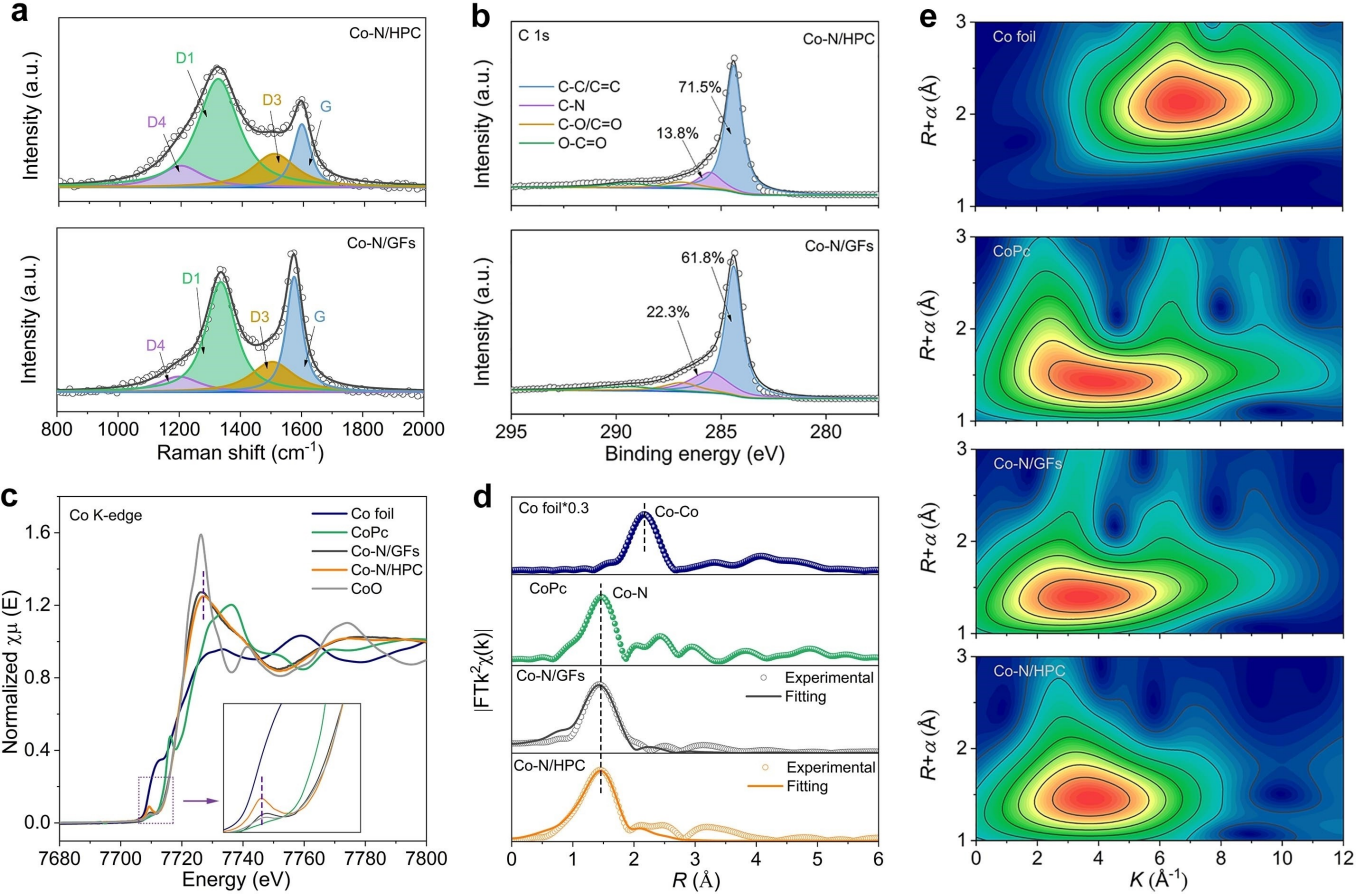
a) Raman spectra of Co−N/GFs and Co−N/HPC. b) High‐resolution C 1s XPS spectra of Co−N/GFs and Co−N/HPC. c) Co K‐edge XANES spectra of different samples (inset: the enlarged image of selected area). d) *k* ^2^‐weighted FT‐EXAFS curves of Co−N/GFs, Co−N/HPC, and reference samples. e) Co K‐edge WT‐EXAFS contour plots of Co foil, CoPc, Co−N/GFs, and Co−N/HPC.

The edge‐ and defect‐rich nature of Co−N/HPC was further verified by Raman spectroscopy. As shown in Figure 3a, the Raman spectra of Co−N/HPC and Co−N/GFs can be deconvoluted into four bands, corresponding to graphitic carbon G‐band (≈1595 cm^−1^) and defect‐related D‐bands: D4 (≈1200 cm^−1^), D1 (≈1320 cm^−1^) and D3 (≈1505 cm^−1^).^[22]^ Compared with Co−N/GFs, the D band peaks of Co−N/HPC are much stronger and broader, indicating a more significant portion of edge defects.^[23]^

The surface chemical states of electrocatalysts were examined by X‐ray photoelectron spectroscopy (XPS). The survey spectra of Co−N/HPC and Co−N/GFs exhibit distinct C, O, and N signals but no detectable Co signal due to its low concentration (Figure S11).^[24]^ The high‐resolution C 1s spectra of samples (Figure 3b) can be fitted by C−C/C=C, C−N, C−O/C=O, and O−C=O peaks, which commonly exist in N‐doped carbon materials.^[25]^ Geometrically, the exposed two N atoms in Co−N_4_/AC‐edge and Co−N_4_/ZZ‐edge models only bond with one carbon atom, while the internal N atoms in the Co−N_4_/G configuration can simultaneously bond with two carbon atoms. Therefore, it is reckoned that Co−N_4_ moieties at the armchair and zigzag edges should exhibit less C−N than the basal‐plane counterpart.^[11]^ The C−N content of Co−N/HPC (13.8 %) is smaller than that of Co−N/GFs (22.3 %), experimentally verifying more edge‐sited Co moieties in Co−N/HPC. The high‐resolution N 1s spectra (Figure S12–13) can be deconvoluted into pyridinic N, pyrrolic N, graphitic N, and oxidized N. The N and O contents are similar for Co−N/HPC and Co−N/GFs, excluding the possible contribution of N and O dopants for varied ORR performances.

Synchrotron‐based X‐ray absorption spectroscopy (XAS) was used to probe the valence states and local structure of Co species. As illustrated in Co K‐edge X‐ray absorption near edge structure (XANES) spectra (Figure 3c), the absorption edges of Co−N/HPC and Co−N/GFs are close to CoO, suggesting the positively charged Co atoms. The noted pre‐edge peak at about 7710 eV corresponds to the 1s→3d transition of Co, indicative of distorted *D*_4h_ symmetry of central Co sites.^[26]^ Obviously, a higher pre‐peak intensity of Co−N/HPC is observed than that of Co−N/GFs, implying decreased symmetry of Co sites due to the edge‐ and pore‐induced local spatial distortion.^[15]^ In addition, the relatively weak white line intensity of Co−N/HPC further indicates a defective graphene architecture around Co sites.[^26^, ^27^] The Fourier transformed extended X‐ray absorption fine structure (FT‐EXAFS) spectra (Figure 3d) show a prominent peak at 1.45 Å in Co−N/HPC and 1.43 Å Co−N/GFs, which can be assigned to the Co−N coordination according to the spectrum of cobalt phthalocyanine (CoPc).^[28]^ Compared with Co foil, the absence of significant long‐range order further reveals the atomic dispersion of Co moieties, in agreement with the TEM observation. The EXAFS wavelet transform (WT) analysis (Figure 3e) shows that the intensity maximum of Co−N/HPC and Co−N/GFs is close to that of CoPc (≈4.0 Å^−1^).^[29]^ The intensity maximum of Co−Co coordination in Co foil (≈6.8 Å^−1^) is not observed in Co−N/HPC and Co−N/GF.^[30]^ The results suggest that the first shell of Co atoms can be assigned to the Co−N scattering path. FT‐EXAFS fitting of Co−N/HPC and Co−N/GFs suggests that the central Co atom is averagely bonded with four N atoms to form the Co−N_4_ coordination (Figure S14–16, Table S2). To further determine the atomic structures, XANES simulations were performed (Figure S17–18). The experimental XANES spectra are more similar to the theoretical spectra simulated based on the proposed Co−N_4_ configurations, confirming the predominance of Co−N_4_ moieties in the prepared catalysts.

**Figure 4:**
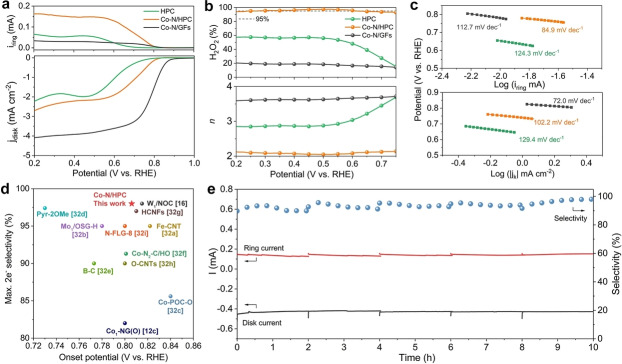
a) LSV curves recorded on the RRDE in O_2_‐saturated 0.1 M KOH at a rotation speed of 1600 rpm. b) Calculated 2 e^−^‐ORR selectivity and electron transfer number (*n*) as a function of applied potentials. c) Tafel plots derived from LSV curves. d) Comparison of 2 e^−^‐ORR onset potential and maximum selectivity in alkaline media between Co−N/HPC and previously reported catalysts (the source references are indicated in the brackets). e) Stability evaluation of Co−N/HPC via chronoamperometric test at a fixed disk potential of 0.50 V vs. RHE.

The electrocatalytic ORR performances of the as‐prepared samples were evaluated in O_2_‐saturated 0.1 M KOH using a rotating ring‐disk electrode (RRDE). The Pt ring electrode was held at a constant potential of 1.2 V vs. reversible hydrogen electrode (RHE) to quantify the amount of generated H_2_O_2_. The collection efficiency was calibrated by the redox reaction of [Fe(CN_6_)]^4−^/[Fe(CN_6_)]^3−^ (Figure S19). The linear sweep voltammetry (LSV) curves (Figure 4a) present the current signals for oxygen reduction and H_2_O_2_ oxidation recorded on the disk electrode and ring electrode, respectively. Sharply different ORR performances are observed for the as‐prepared catalysts. Specifically, the Co−N/GFs catalyst presents a typical quasi‐4 e^−^ pathway with the earliest onset potential, the highest diffusion‐limiting disk current density, and the lowest ring current. In contrast, the highest ring current is observed on Co−N/HPC, suggesting the generation of more H_2_O_2_ during the ORR process. The calculated 2 e^−^ selectivity and electron transfer number (*n*) were plotted in Figure 4b as a function of applied potentials. The average 2 e^−^‐ORR selectivity of Co−N/HPC is about 95 %, with a maximum value of 98 % at 0.52 V (vs. RHE). The corresponding electron transfer number is below 2.1 in a wide potential range of 0.2–0.75 V (vs. RHE), indicating a highly selective 2 e^−^‐ORR pathway. The calculated turnover frequency of Co−N/HPC is significantly higher than that of Co−N/GFs (Figure S20), indicating better catalytic efficiency towards 2 e^−^‐ORR. In the absence of single‐atomic Co sites, the 2 e^−^‐ORR selectivity of HPC reaches 58 %, while the activity is relatively low, as reflected by the large overpotential. The significantly enhanced catalytic activity and selectivity of Co−N/HPC suggest the superior reactivity of Co−N_4_ moieties towards 2 e^−^‐ORR. The Tafel plots were calculated to analyze the reaction kinetics (Figure 4c and Figure S21). The slope of Co−N/HPC from the disk electrode (102.2 mV dec^−1^) is larger than that of Co−N/GFs (72.0 mV dec^−1^), suggesting that the ORR process is co‐dominated by OOH* formation and further dissociation.[^12b^, ^31^] In contrast, the slope of Co−N/HPC from the ring electrode is the lowest (84.9 mV dec^−1^) among all the catalysts, suggesting the fastest H_2_O_2_ generation kinetics.^[12b]^ The comparison of 2 e^−^‐ORR catalytic performance between Co−N/HPC and previously reported state‐of‐the‐art catalysts were provided in Figure 4d.[^12c^, ^16^, ^32^] As can be seen, the Co−N/HPC catalyst exhibits comparable catalytic activity to most SACs and the best catalytic selectivity among them. Similar activity and selectivity trends of Co−N/HPC and Co−N/GFs can also be observed in the acidic media (Figure S22). Moreover, Co−N/HPC displays excellent catalytic stability, as evidenced by negligible decay in the ring and disk currents during the 10 h chronoamperometric test. The corresponding selectivity remains above 90 %.

Notably, the intrinsic defect and edge structures in carbon materials could also act as potential catalytic sites for ORR.^[33]^ To identify the catalytic site, the poison experiment was carried out by using SCN^−^ ions, which tend to bind the metal atoms of M−N−C SACs and block the adsorption of reaction intermediates, thus negatively affecting the catalytic performance.^[34]^ The significant degradation of the catalytic activity after adding SCN^−^ into the electrolyte strongly verifies that the ORR occurs predominantly on the single‐atomic Co−N_4_ sites (Figure S23).

The electrochemically active surface areas (ECSA) were evaluated by measuring the double‐layer capacitances (*C*_dl_) of catalysts. As shown in Figure S24, the *C*_dl_ value of Co−N/HPC (13.2 mF cm^−2^) is slightly higher than that of Co−N/GFs (11.1 mF cm^−2^). This suggests higher availability of exposed active sites within the HPC (i.e., edges). Considering the analogous elemental composition of Co−N/HPC and Co−N/GFs, the substantial variation in their catalytic behaviors can be attributed to the different locations of Co−N_4_ sites within the carbon matrix. The HPC with rich in‐plane pores and edges enables the formation of Co−N_4_ moieties over these defective sites, which are more favorable for the 2 e^−^‐ORR pathway than basal‐plane‐hosted ones. To provide further evidence, we increased the metal concentration during the synthesis procedure, and it was found that the 2 e^−^‐ORR selectivity dropped dramatically on the resultant sample (Figure S25). Alternatively, the 4 e^−^‐ORR pathway became dominant. This is because the increased metal loading will lead to more Co−N_4_ sites in the basal plane, thus shifting the ORR towards the 4 e^−^ route. These results suggest that Co−N_4_ moieties with varied locations in the carbon substrate contribute to the different ORR catalytic reactivity observed on Co‐SACs.

The catalytic performance for the scale‐up H_2_O_2_ production was further evaluated in a customized H‐type cell (Figure S26). The chronoamperometric test (Figure S27) shows that Co−N/HPC loaded electrode can deliver a current density of ≈10 mA cm^−2^ at 0.5 V (vs. RHE). Upon continuous operation for 10 h, the H_2_O_2_ production rate can be up to 1.72 mol h^−1^ g_cat_^−1^ as determined by the Ce^4+^/Ce^3+^ colorimetric method (Figure S28), with high Faradaic efficiency (FE) of 92.3 %.^[12b]^ The H_2_O_2_ yield of Co−N/HPC in bulk electrocatalysis is comparable and even superior to the recently reported 2 e^−^‐ORR electrocatalysts in the literature (Table S3).

**Figure 5:**
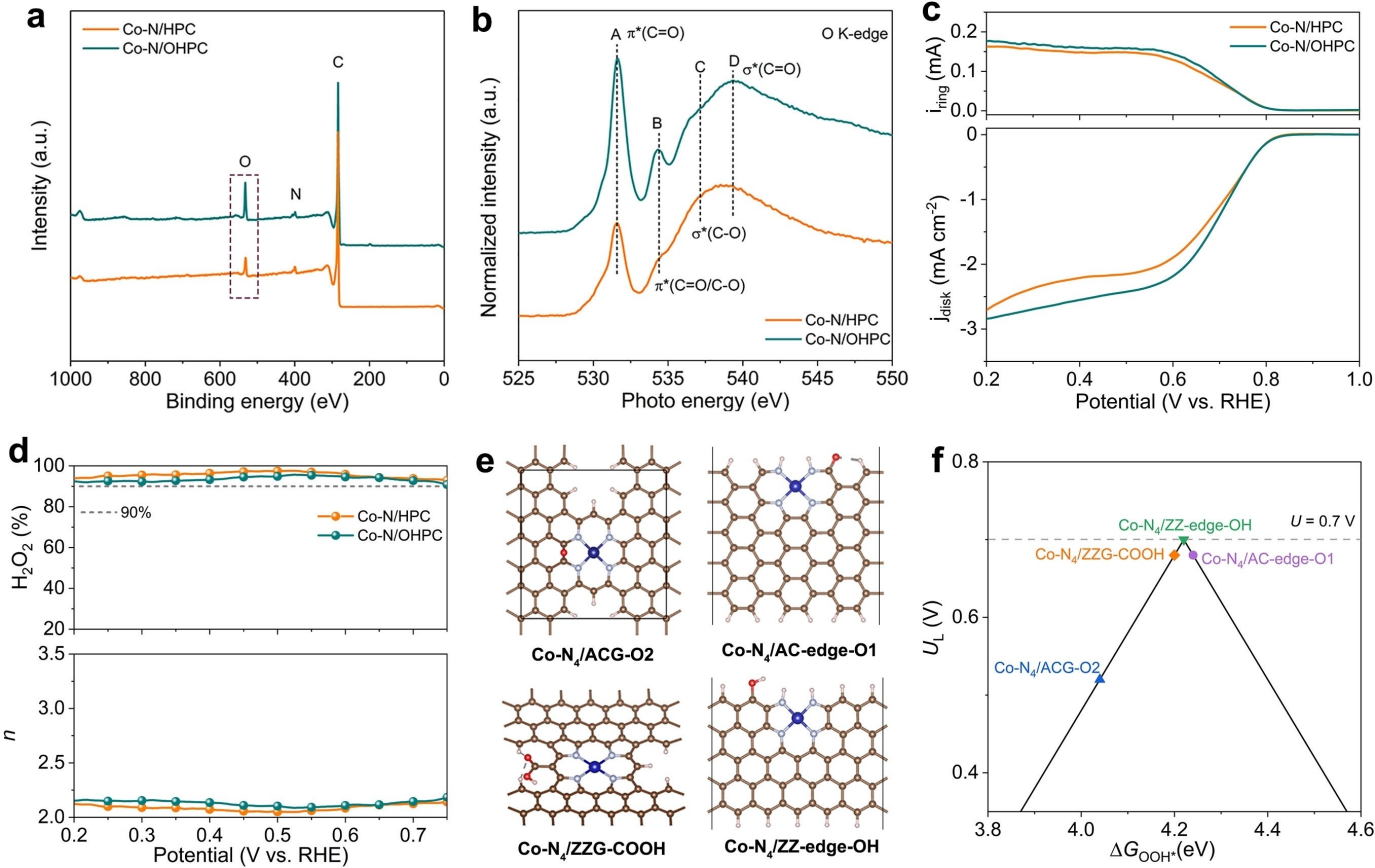
a) XPS survey spectra and b) O K‐edge XANES spectra of Co−N/HPC and Co−N/OHPC. c) LSV curves of Co−N/HPC and Co−N/OHPC recorded on RRDE in O_2_‐saturated 0.1 M KOH. d) Calculated 2 e^−^ selectivity and electron transfer number (*n*) as a function of applied potentials. e) Optimized structural models of Co−N_4_/ACG−O2, Co−N_4_/AC‐edge‐O1, Co−N_4_/ZZG‐COOH and Co−N_4_/ZZ‐edge‐OH. Color code: the brown, grey, red, blue, and pink balls refer to C, N, O, Co, and H atoms, respectively. f) Corresponding volcano plot for the 2 e^−^‐ORR.

For carbon‐based 2 e^−^‐ORR electrocatalysts, the generated H_2_O_2_ would attack the carbon matrix directly and/or indirectly through the decomposition of H_2_O_2_ into more oxidative reactive oxygen species (i.e., hydroxy and hydroperoxy radicals).^[35]^ This oxidative corrosion would result in a series of OFGs on the carbon surface and affect the catalytic performance.^[32c]^ To examine the compositional/structural changes after the bulk electrocatalysis, we collected the used Co−N/HPC catalyst (denoted as Co−N/OHPC) for post‐catalysis characterizations. The original morphology and defect nature are inherited by Co−N/OHPC, as confirmed by the SEM image (Figure S29) and Raman spectrum (Figure S30). However, the XPS survey (Figure 5a) scan shows an increased O concentration from 4.0 at.% for Co−N/HPC to 7.6 at.% for Co−N/OHPC.^[12d]^ The increased proportion of oxygen species is also evident in the C 1s spectrum of Co−N/OHPC (Figure S31), while the N 1s spectrum is less affected (Figure S32).

The synchrotron‐based soft XAS was conducted to discriminate the oxygen species attached to the catalyst. In the O K‐edge XANES spectra (Figure 5b), peak A at ≈531.8 eV is assigned to the C=O group contributed by ketones and/or carboxylic acids.^[36]^ Peak B at ≈534.3 eV results from the 1s→π* excitation due to the charge transfer between C and O (including C=O and C−O).^[36b]^ Peak C at ≈537.2 eV and peak D at ≈539.6 eV can be attributed to the σ* C−O and σ* C=O resonances, respectively.^[37]^ As can be seen, the peak signals of OFGs are more pronounced in Co−N/OHPC, providing unambiguous evidence for the formation of OFGs during ORR operation. This result is also consistent with the previous findings.^[38]^ The comparison of Co K‐edge XANES spectra between Co−N/OHPC and Co−N/HPC was provided in Figure S33a. The absorption edge position and the white line intensity slightly increase, which may be attributable to the electron‐withdrawing effect of attached oxygen species around the Co atoms.[^12d^, ^39^] The corresponding FT‐EXAFS spectrum confirms the well‐retained atomic dispersion of Co sites (Figure S33b).

The 2 e^−^‐ORR activity and selectivity of oxygen‐functionalized Co−N/OHPC were further examined. As shown in Figure 5c, increased disk and ring currents are observed for Co−N/OHPC. The selectivity drops slightly compared to Co−N/HPC but remains above 90 % (Figure 5d). The Tafel slopes of Co−N/OPHC are smaller than these of Co−N/HPC (Figure S34). Furthermore, the mass activity increases from 41.8 A g_cat_^−1^ of Co−N/HPC to 67.7 A g_cat_^−1^ of Co−N/OHPC. These results demonstrate the promoted ORR catalytic kinetics and activity induced by formed OFGs on the carbon matrix.

Following the experimental investigations, DFT calculations were further employed to understand how OFGs affect the ORR catalytic activity and selectivity. Different types of OFGs, including hydroxyl (−OH), carbonyl (C=O), epoxy (C−O−C), and carboxyl (−COOH), were introduced beside the Co−N_4_ sites in Co−N_4_/ACG, Co−N_4_/AC‐edge, Co−N_4_/ZZG, and Co−N_4_/ZZ‐edge models (a total of 16 configurations in Figure S35). The edge sites are subject to the modification of OFGs owing to their more reactive nature than basal carbons.^[23]^ By calculating the Δ*G*_OOH*_ on various configurations (Figure S36), it is found that the Co−N_4_/ACG−O2 with the epoxy group, the Co−N_4_/AC‐edge‐O1 with the carbonyl group, and the Co−N_4_/ZZG‐COOH with the carboxyl group have more suitable Δ*G*_OOH*_ values and demonstrate high catalytic activities (Figure 5f). Remarkably, the Co−N_4_ site at the −OH functionalized zigzag edge (Co−N_4_/ZZ‐edge‐OH), sitting at the top of the volcano, possesses zero overpotential and the highest catalytic activity toward the 2 e^−^‐ORR. Moreover, introducing the epoxy group adjacent to the basal‐plane‐hosted Co−N_4_ sites can also considerably weaken the binding strength of the OOH* intermediate (Figure S37).

Based on the above analyses, it can be concluded that the nearby OFGs can further adjust the binding strength of OOH* over Co−N_4_ sites toward more favorable 2 e^−^‐ORR energetics.[^12d^, ^40^] According to previous reports, the carbon sites at the oxygen‐terminated edges could be activated as additional binding sites to promote the electrochemical synthesis of H_2_O_2_ via 2 e^−^‐ORR process.[^22a^, ^38a^] As a result, the synergy between generated OFGs and Co−N_4_ sites on the edge‐rich Co−N/HPC contributes to the facilitated reaction kinetics, enhanced catalytic activity, and well‐maintained selectivity. These findings suggest that the location of active sites and surrounding functional groups are critical factors in regulating the catalytic activity and selectivity of SACs. Strategies to engineer the carbon matrix can finely tune the local coordination environment of the atomic site for desirable catalytic properties. The contribution of structural changes of SACs under reaction conditions should be considered for probing the assignment of actual active sites.

## Conclusion

In summary, we have elucidated the prominent role of the edge structures in controlling the ORR pathways (i.e., 2 e^−^ and 4 e^−^) on Co‐SACs. The theoretical calculations suggest that the edge‐hosted Co−N_4_ sites (i.e., atomic Co−N_4_ moieties residing in the defective pockets and edges) exhibit highly selective 2 e^−^‐ORR catalytic activity, while the basal‐plane‐hosted sites contribute to the 4 e^−^‐ORR. As a proof‐of‐concept, we have prepared carbon‐supported Co‐SACs with various edge‐to‐bulk ratios. Remarkably, around 95 % selectivity with a high H_2_O_2_ production rate (1.72 mol h^−1^ g_cat_^−1^) and a high FE (>90 %) is achieved on the edge‐rich Co−N/HPC catalyst. More importantly, we have observed that the exposed edges could be readily oxygenated with oxygen functional groups during the ORR process, which promotes the 2 e^−^‐ORR kinetics and maintain the selectivity above 90 %. Our findings highlight the fine‐tuning of anchoring sites and functional groups for regulating the ORR pathway on SACs and could provide an inspiring route to realizing large‐scale and low‐cost electrochemical H_2_O_2_ production.

## Conflict of interest

The authors declare no conflict of interest.

1

## Supporting information

As a service to our authors and readers, this journal provides supporting information supplied by the authors. Such materials are peer reviewed and may be re‐organized for online delivery, but are not copy‐edited or typeset. Technical support issues arising from supporting information (other than missing files) should be addressed to the authors.

Supporting Information

## Data Availability

The data that support the findings of this study are available from the corresponding author upon reasonable request.
