# Supplementary material for: Edge‐hosted Atomic Co−N4 Sites on Hierarchical Porous Carbon for Highly Selective Two‐electron Oxygen Reduction Reaction
Source: Angew Chem Int Ed Engl. 2022 Nov 17;61(51):e202213296. doi: 10.1002/anie.202213296 (PMC10098864; doi:10.1002/anie.202213296)
Supplement: Supplementary file 1 — Supporting Information [file ANIE-61-0-s001.pdf]

Angewandte Chemie
Eine Zeitschrift der Gesellschaft Deutscher Chemiker

Supporting Information

Edge-hosted Atomic Co–N_4 Sites on Hierarchical Porous Carbon for Highly Selective Two-electron Oxygen Reduction Reaction

Y. Tian, M. Li, Z. Wu, Q. Sun, D. Yuan, B. Johannessen, L. Xu, Y. Wang, Y. Dou, H. Zhao, S. Zhang**

Experimental section

Material synthesis

The honeycomb-like hierarchical porous carbon (HPC) was synthesized according to a previous report with minor modifications.[1] In a typical synthesis, NaCl and coffee extract were first dissolved into deionized (DI) water with a mass ratio of 5:1. After freeze-drying, the acquired coffee-NaCl solid mixture was calcinated at 400 °C under Ar atmosphere for 1 h and then at 950 °C for another 1 h with a ramping rate of 2 ℃ min^{-1}. Next, the residual NaCl was then removed by rinsing and filtering with DI water for several times. Finally, the powders were obtained after drying in a vacuum oven at 60°C overnight. The comparison matrix of graphite flakes (GFs) was prepared by ultrasonication in the sequence of diluted hydrochloric acid, acetone, ethanol, and H_2O for 1 h, respectively, and then dried in a vacuum oven at 60 ºC overnight.

The Co-N/HPC catalyst was prepared via a wet impregnation method. Typically, 23.8 mg of $CoCl_2{\cdot}6H_2O$ and 36 mg of 1,10-phenanthroline (Phen) were first dissolved in 100 mL ethanol to form the Co-Phen complex at room temperature. Then, 50 mg of HPC was immersed into the above solution and constantly stirred at 60 °C for 3 h. After repeated washing and filtering, the Co-Phen impregnated HPC was dried in a vacuum oven and then calcined at 900 ℃ for 1 h under Ar atmosphere. Next, the obtained powders were etched by 1 M HCl to remove surface metal particles and unstable species. Finally, the Co-N/HPC catalyst was obtained after further heat treatment at 800 ℃ for 15 min under vacuum to remove residual anions or impurities absorbed on the active sites during the etching process. The Co-N/HPC catalyst with a higher Co loading was synthesized by adding 119 mg of $CoCl_2{\cdot}6H_2O$ and 180 mg of 1,10-phenanthroline. The preparation of the Co-N/GFs catalyst follows the same procedure described above.

The Co-N/OHPC catalyst was obtained by oxidizing the Co-N/HPC sample through bulk H_2O_2 production in 0.1 M KOH. The sample was rinsed with deionized water and ethanol several times and then dried in a vacuum oven at 60 °C before further characterizations.

Physical characterizations: Field-emission scanning electron microscopy (JEOL JSM-7800F) and transmission electron microscopy (JEOL JEM-2010F) were utilized to observe the morphology of the prepared samples. The atomic-resolution STEM images were obtained by JEOL JEM-ARM200F. The crystal structures were

characterized by X-ray diffraction (Shimadzu, XRD-6100) using a high-intensity Cu Kα radiation source (λ=1.54 Å) and operating at a voltage of 40 kV and a current of 30 mA. X-ray photoelectron spectroscopy (XPS) analysis was performed using a Thermo ESCALB 250XI X-ray photoelectron spectrometer. Co K-edge X-ray absorption spectra were collected on the hard X-ray beamline at the Australian Synchrotron (Melbourne, Australia). The extended X-ray absorption fine structure (EXAFS) analyses were conducted via the ATHENA module. Fourier transform of the k^2-weighted EXAFS oscillations was used to evaluate the contribution of each bond to the Fourier transform peak.

Electrochemical measurements: All electrochemical measurements were performed on a CHI 760E electrochemical workstation (CH Instrument) coupled with a rotating-ring disc electrode (RRDE) in a three-electrode cell. A graphite rod and an Ag/AgCl electrode with saturated KCl were used as the counter and reference electrodes, respectively. To prepare the working electrode, 2 mg of catalyst powder was dispersed into 1.0 mL of 1:1 water/ethanol solution and 20 μL of Nafion solution (10 wt.%), followed by ultrasonic treatment. Then, the catalyst ink was cast on the surface of RRDE with a catalyst loading of ~0.1 mg cm^{-2}. All the potentials were calibrated to the reversible hydrogen electrode (RHE) according to the Nernst equation (Eqn. 1)

$$E_{\mathrm{RHE}} = E_{\mathrm{Ag/AgCl}} + 0.059pH + 0.197 \qquad (1)$$

For ORR testing, LSV curves were recorded in O_2-saturated 0.1 M KOH with a scan rate of 5 mV s^{-1} at a rotating speed of 1600 rpm. The capacitive current was eliminated by deducting the background current in the N_2-saturated electrolyte. The ring electrode potential was set to 1.20 V (vs. RHE) to oxidize generated H_2O_2 from the disk electrode. The electron transfer number and the selectivity were calculated according to Eqns. 2–3:

$$n = 4|I_d|/(|I_d| + I_r/N) \qquad (2)$$

$$\text{Selectivity (\%)} = 200 \times I_r / (I_r + N|I_d|) \qquad (3)$$

where I_d is the disk current; I_r is the ring current; N is the current collection efficiency of the Pt ring with a value of 0.3769, which was determined by the $[Fe(CN)_6]^{3-/4-}$ redox couple (Figure S19).

The kinetic current density (j_k) was calculated to the equation:

$$1/j_m = 1/j_L + 1/j_k \qquad (4)$$

where j_m indicates the measured total current density and j_L is the diffusion-limiting current density. The value of j_L is usually determined by the highest steady current measured in the entire potential range.

The turnover frequency (TOF) was calculated based on the following equation:

$$\mathrm{TOF} = \frac{I_{H_2O_2} M_{Co}}{nFM_{cat}\omega_{Co}} \qquad (5)$$

where I_{H2O2} is the current for H_2O_2 production; M_{Co} is the molar mass of cobalt (58.933 g mol^{-1}); n is the number of transferred electrons based on RRDE results; F is the Faraday constant (96485 C mol^{-1}); M_{cat} is the mass of catalyst on the electrode; ω_{Co} is the mass ratio of Co in the catalyst.

Tafel slopes were calculated from the Tafel equation:

$$\eta = \mathrm{b}\log(j/j_0) \qquad (6)$$

where η is the potential, b is the Tafel slope, j is the current density, and j_0 is the exchange current density.

The double-layer capacitances (C_{dl}) of different catalysts were calculated by recording different CV curves at a variety of sweep rates of 5, 10, 15, 20, and 25 mV s^{-1} in the potential range of 0.9–1.0 V (vs. RHE). The chronoamperometry test was performed at 0.5 V (vs. RHE).

The bulk electrosynthesis of H_2O_2 in 0.1 M KOH was carried out in a customized H-cell electrolyzer. The catalyst ink was drop-casted onto the carbon paper electrode (reaction area = 1×1 cm^2) as the working electrode. A Nafion 117 membrane was employed to separate the anode and cathode chambers. The working electrode and reference electrode (Ag/AgCl) were placed in the cathode chamber, while the Ni foam was loaded in the anode chamber as the counter electrode. Both chambers were filled with 70 mL 0.1 M KOH. The cathode chamber was bubbled with pure O_2 during the electrolytic process. The concentration of generated H_2O_2 was determined by the cerium sulfate $Ce(SO_4)_2$ titration method based on the mechanism that yellow-colored Ce^{4+} transforms to colorless Ce^{3+} after reaction with H_2O_2 ($2Ce^{4+} + H_2O_2 \rightarrow 2Ce^{3+} + 2H^+ + O_2$). Absorption spectroscopy of standard Ce^{4+} solutions was performed on an ultraviolet-visible (UV-vis) spectrometer (Cary-300 Series, Agilent Technologies) at 319 nm. The Ce^{4+} concentration-absorbance curve was plotted by linearly fitting the absorbance values at a wavelength length of 319 nm for known concentrations of 0,

0.025, 0.05, 0.1, 0.25, and 0.5 mM of Ce^{4+}. The obtained sample solutions were mixed with 0.5 mM Ce^{4+} solution by specific dilution. The stock solution was rested for 2 h before UV-vis measurement to ensure the completion of the reaction of H_2O_2. The concentration of remaining Ce^{4+} in the electrolyte can be calculated using the linear fitted equation. The H_2O_2 yield was determined based on the consumed Ce^{4+} concentration. The Faradaic efficiency (FE) for H_2O_2 generation in H-cell was calculated as follows:

$$\mathrm{FE}\ (\%) = 100 \times \frac{n \times F \times \text{Cumulative } \mathrm{H_2O_2} \text{ yield (mol)}}{\int_0^t I\ \mathrm{d}t} \qquad (7)$$

where n is the electron transfer number (2 for the dioxygen reduction into H_2O_2); F is the Faraday constant (96485.3 C mol^{-1}); $\int_0^t I\ \mathrm{d}t$ is the consumed quantity of electric charge (C).

Theoretical calculations

The density functional theory (DFT) calculations were performed in the Vienna ab initio simulation packages (VASP),[2] using the generalized gradient approximation (GGA) with Perdew–Burke–Ernzerhof (PBE) parameterization.[3] According to previous reports,[4] the Co–N_4 unit-embedded monolayer graphene structures were adopted for simulation. A vacuum space of 20 Å was set to eliminate possible interaction between the periodic images. The energy cut-off of the plane wave basis was set as 520 eV. The semi-empirical dispersion corrected DFT-D3 scheme proposed by Grimme was used to describe the van der Waals interactions.[5] Spin-polarization was applied for all the calculations. During the geometry optimization, the energy change criterion was set to 10^{-5} eV, and the maximum force was 0.03 eV $Å^{-1}$.

The formation energy (E_{form}) of the atomic Co–N_4 moiety is defined as:

$$E_{\mathrm{form}} = E_{\mathrm{Co@gra}} - E_{\mathrm{gra}} - E_{\mathrm{Co}} \qquad (8)$$

where $E_{\mathrm{Co@gra}}$ E_{gra}, and E_{Co} represent the total energies of single atom configuration, graphene substrate, and Co atom in the bulk phase. According to this definition, the more negative formation energy indicates the thermodynamically more favorable to stabilize atomic Co–N_4 moiety.

The two-electron ORR process can be described below (Eqns. 9–10):

$$O_2(g) + * + H^+ + e^- \leftrightarrow OOH* \quad (9)$$

$$OOH* + e^- + H^+ \leftrightarrow * + H_2O_2 \quad (10)$$

The four-electron ORR process can be described as follows (Eqns. 11–14):

$$O_2(g) + * + H^+ + e^- \leftrightarrow OOH* \quad (11)$$

$$OOH* + H^+ + e^- \leftrightarrow O* + H_2O \quad (12)$$

$$O* + H^+ + e^- \leftrightarrow OH* \quad (13)$$

$$OH* + H^+ + e^- \leftrightarrow * + H_2O \quad (14)$$

where * represents an active site on the catalyst surface.

The free energies of ORR/OER intermediates are defined as $\Delta G = \Delta E + \Delta ZPE - T\Delta S$, where ΔE, ΔZPE, T, and ΔS represent the reaction energy, zero-point energy, temperature (298.15 K), and the entropy, respectively.

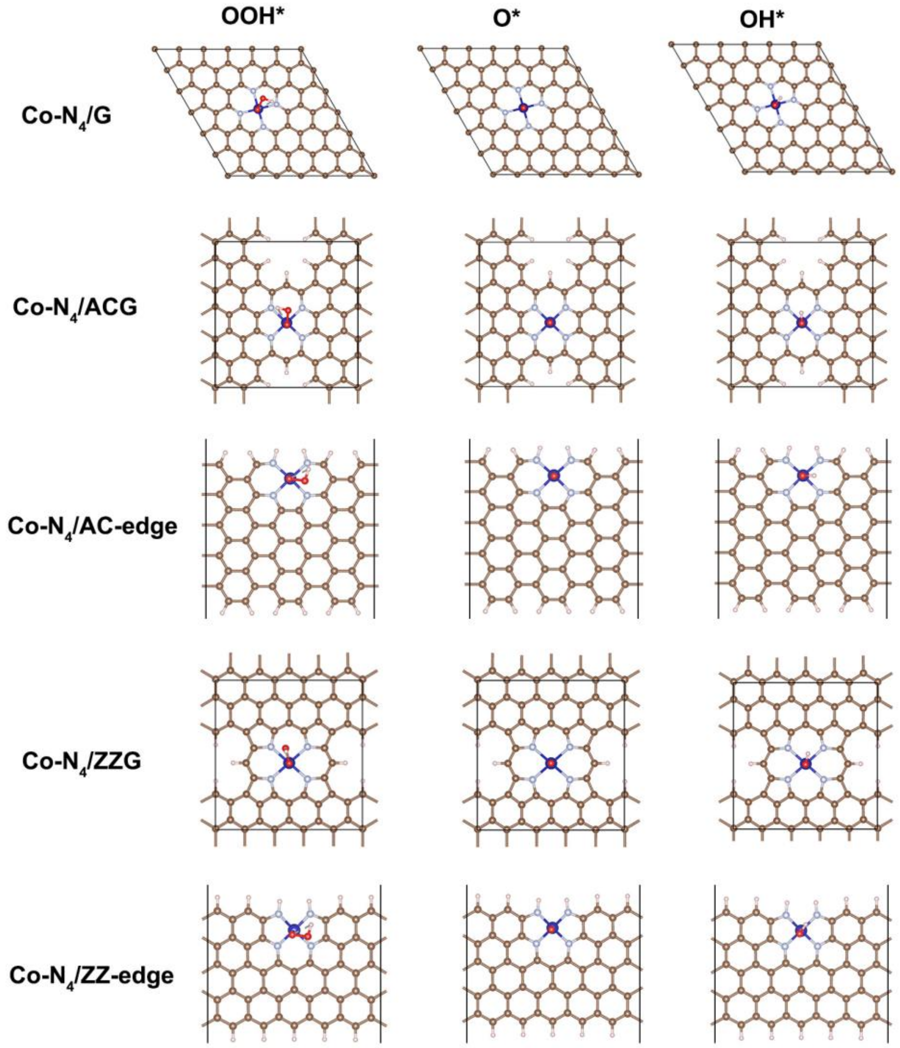

Figure S1. Optimized structural models of different atomic Co–N_4 configurations with ORR intermediates of OOH*, O*, and OH*. Colour code: the brown, grey, blue, red, and pink balls refer to C, N, Co, O, and H atoms, respectively.

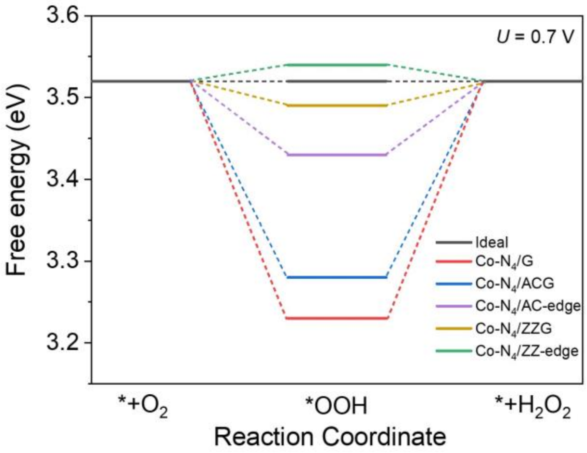

Figure S2. Free energy diagrams of $2e^-$-ORR on the different atomic $Co–N_4$ configurations at U = 0.7 V.

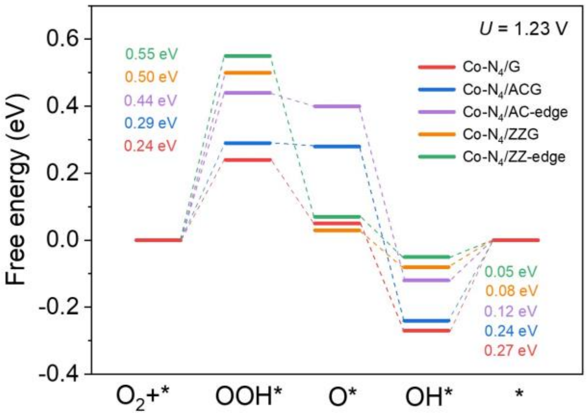

Figure S3. Free energy diagram of the 4e^{-}-ORR on Co–N_4/G, Co–N_4/ACG, Co–N_4/AC-edge, Co–N_4/ZZG, and Co–N_4/ZZ-edge configurations at U = 1.23 V and corresponding energy barriers for OOH* formation and OH* desorption.

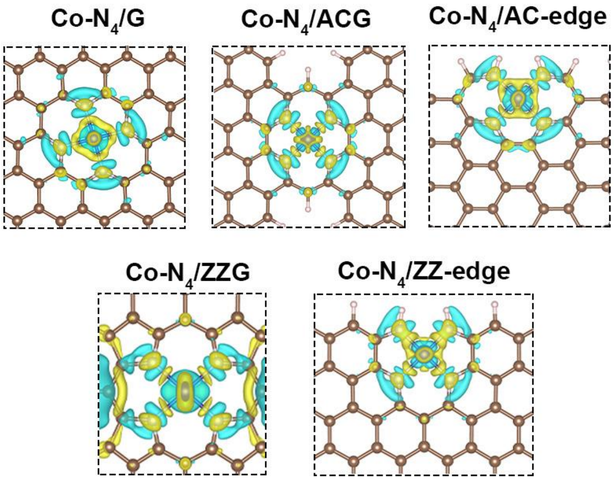

Figure S4. Differential charge densities of different atomic Co–N_4 configurations. The isosurface value is set to be 0.005 eÅ^{-1}. The charge accumulation and depletion areas are shown in yellow and cyan, respectively.

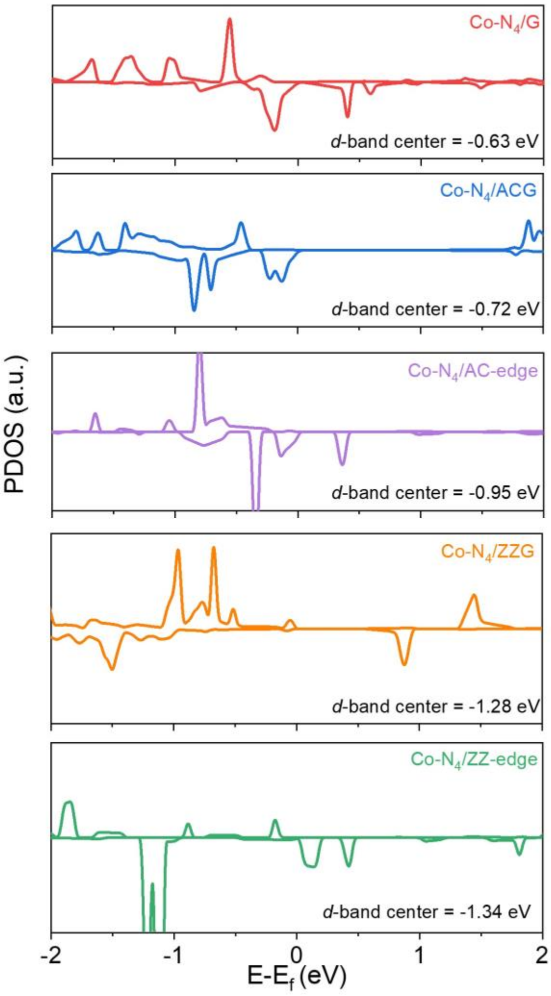

Figure S5. Partial density of states (PDOS) of Co 3d-orbital in different models.

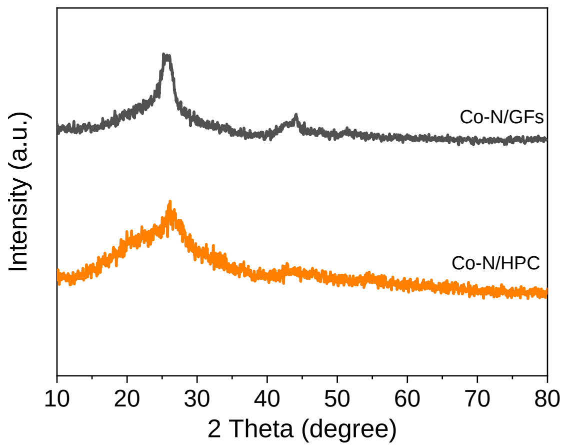

Figure S6. XRD patterns of Co-N/HPC and Co-N/GFs.

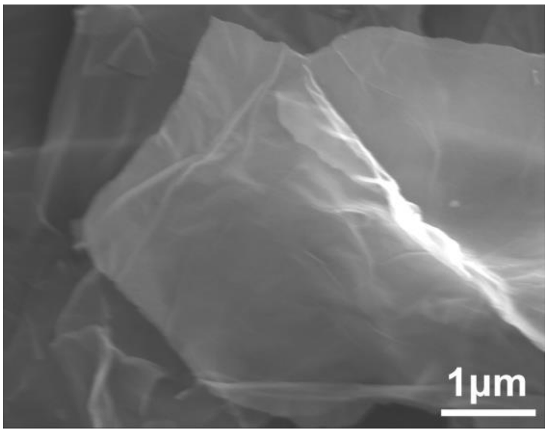

Figure S7. SEM image of Co-N/GFs.

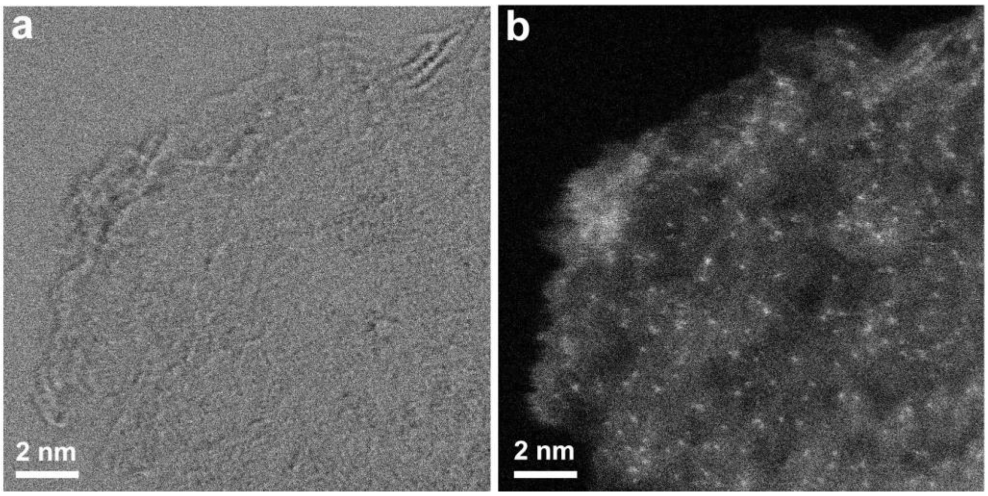

Figure S8. a) High-resolution BF-STEM and b) HAADF-STEM image of Co-N/GFs.

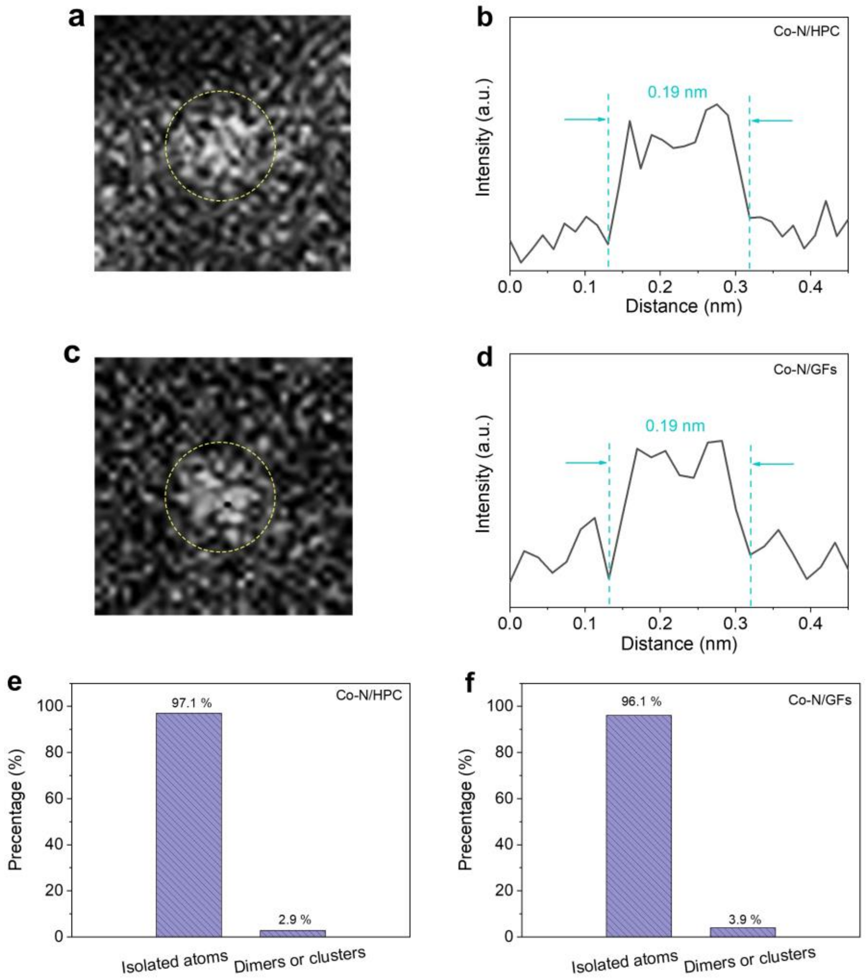

Figure S9. a) Enlarged HAADF-STEM image of an individual bright spot in Co-N/HPC and d) corresponding intensity profile. c) Enlarged HAADF-STEM image of an individual bright spot in Co-N/GFs and d) corresponding intensity profile. Distribution histogram showing the ratio between isolated atoms and dimers or clusters in e) Co-N/HPC and f) Co-N/GFs as determined by counting over 200 bright spots.

The dimers or clusters were determined by the diameter of the bright spot over 0.24 nm.[6] The defect-rich HPC can provide abundant anchoring sites to confine Co ions and effectively prevent their aggregation, resulting in a slightly higher percentage of isolated Co atoms in Co-N/HPC than Co-N/GFs.[7]

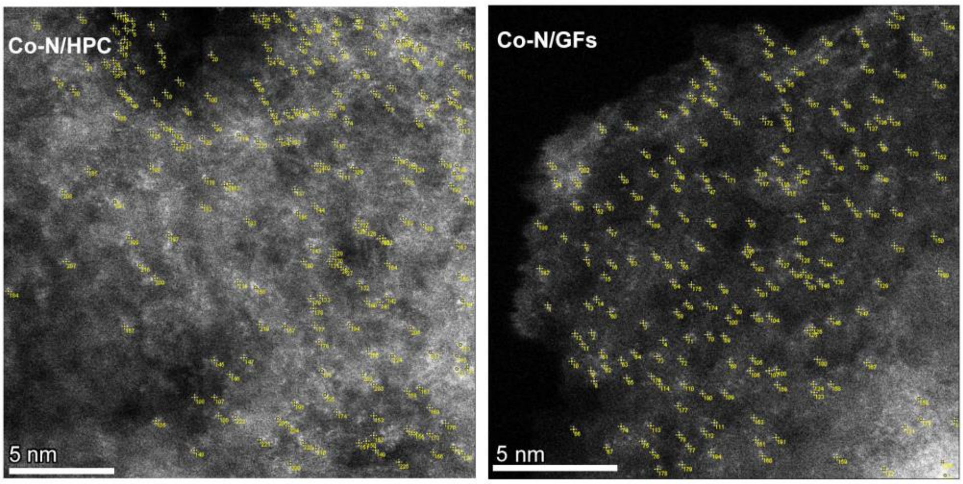

Figure S10. HAADF-STEM images of Co-N/HPC and Co-N/GFs.

The bright spots in the images are marked for quantification of the ratio of the edge-hosted Co sites. It is worth noting that the ratio of edge-hosted sites in Co-N/HPC could be underestimated because some Co atoms at defective pockets and edges may not be visible due to the overlapping of hierarchical carbon layers.

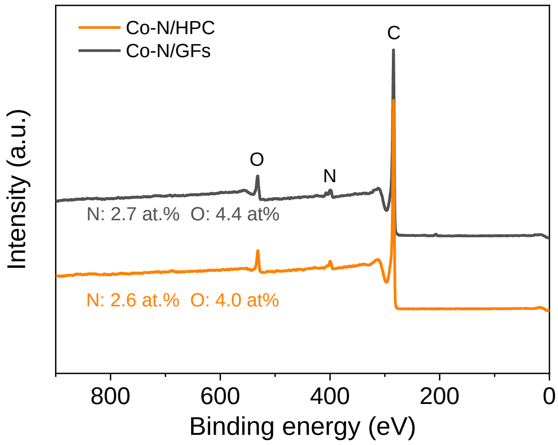

Figure S11. XPS survey spectra of Co-N/HPC and Co-N/GFs.

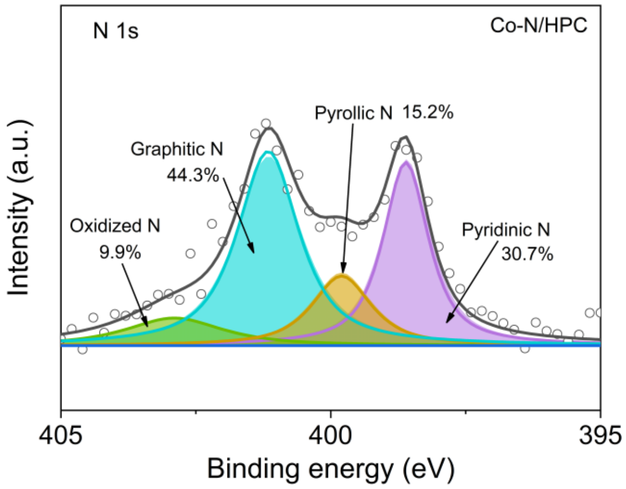

Figure S12. High-resolution N 1s XPS spectrum of Co-N/HPC.

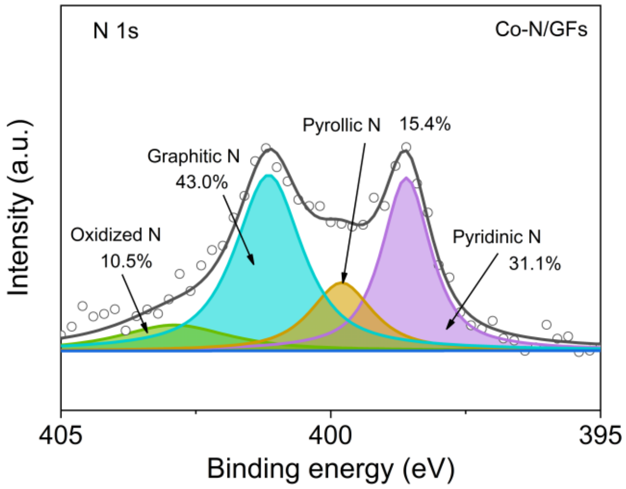

Figure S13. High-resolution N 1s XPS spectrum of Co-N/GFs.

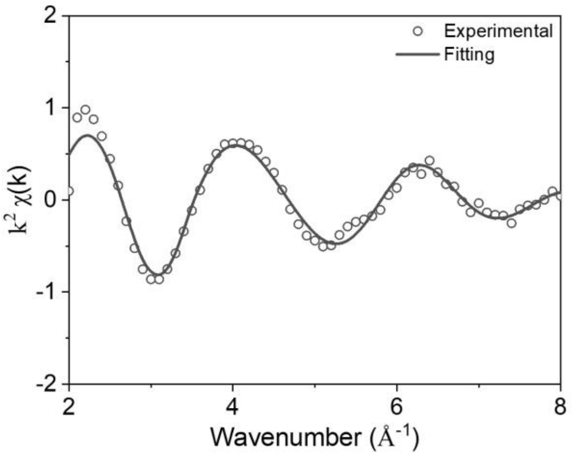

Figure S14. EXAFS fitting of Co-N/GFs at *K* space.

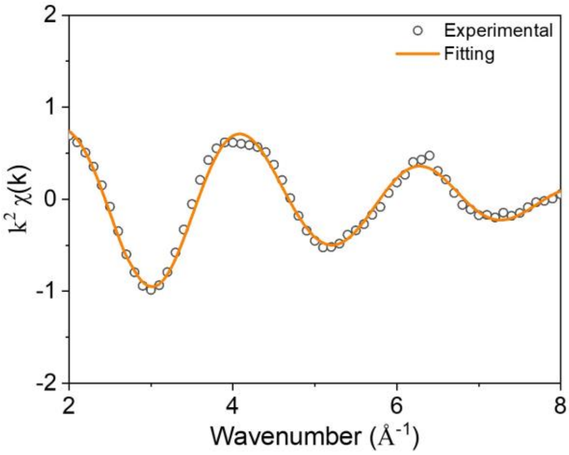

Figure S15. EXAFS fitting of Co-N/HPC at *K* space.

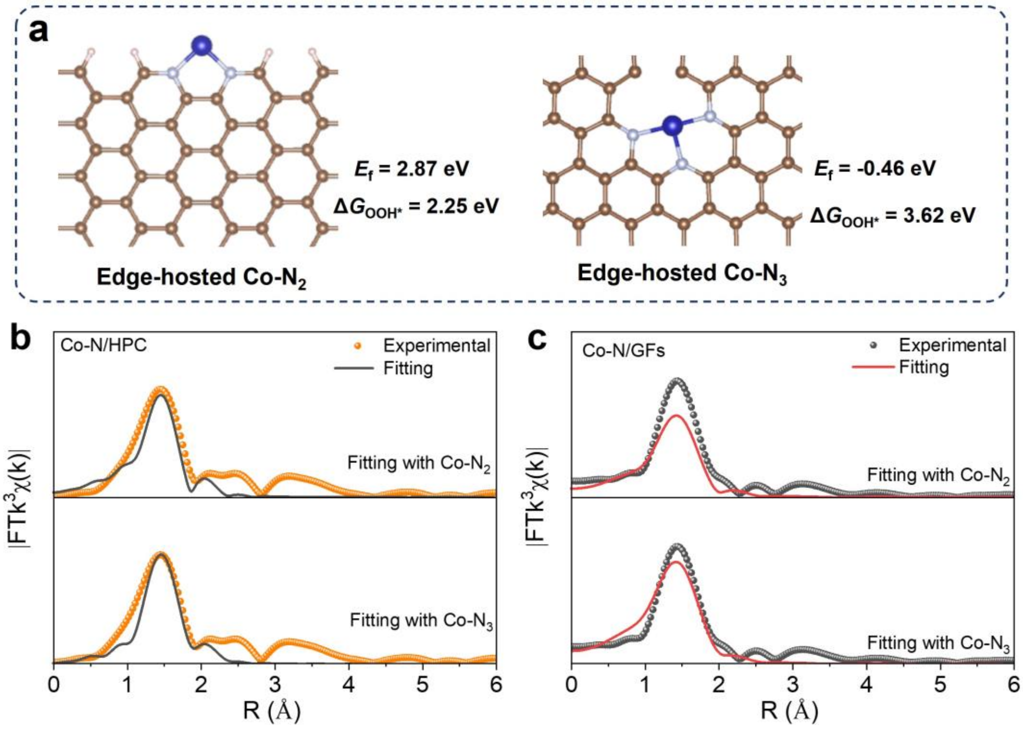

Figure S16. a) Optimized structural models of edge-hosted Co–N_2 and Co–N_3 models and DFT-calculated formation energy (E_f) and ΔG_{OOH^*} (color code: the brown, grey, blue, and pink balls refer to C, N, Co, and H atoms, respectively). Experimental and fitted FT-EXAFS spectra of b) Co-N/HPC and c) Co-N/GFs based on Co–N_2 and Co–N_3 models.

DFT calculations suggest that the formation energies (E_f) of edge-hosted Co–N_2 and Co–N_3 sites are more positive than calculated Co–N_4 sites in Figure 1b. This suggests the inferior stability of edge-hosted Co–N_2 and Co–N_3 sites, which could be etched by the acid solution during the fabrication process. Meanwhile, the calculated ΔG_{OOH^*} values suggest that they are not efficient active sites for $2e^-$-ORR. Overall, these results further support that the $2e^-$-ORR performance is specifically associated with the edge-hosted Co–N_4 moieties.

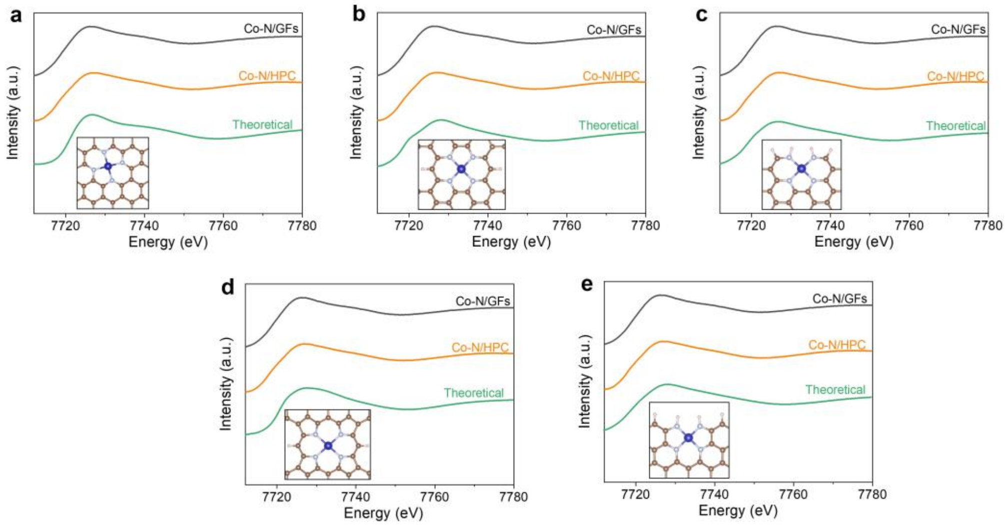

Figure S17. Comparison of the Co K-edge XANES experimental spectra of Co-N/GFs and Co-N/HPC and the theoretical spectra simulated with Co–N_4 configurations including a) Co–N_4/G, b) Co–N_4/ACG, c) Co–N_4/AC-edge, d) Co–N_4/ZZG, and e) Co–N_4/ZZ-edge (color code: the brown, grey, blue, and pink balls refer to C, N, Co, and H atoms, respectively).

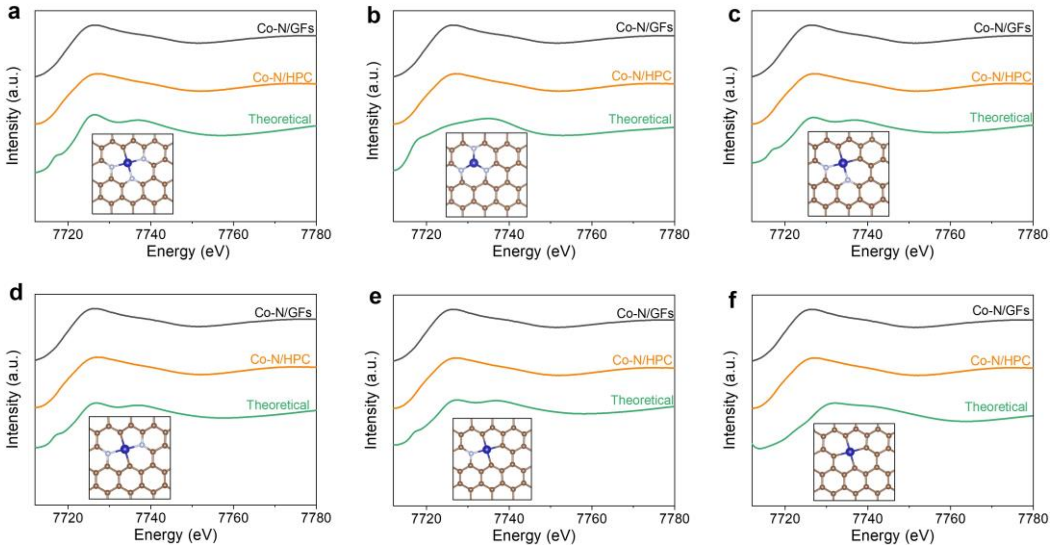

Figure S18. Comparison of the K-edge XANES experimental spectra of Co-N/GFs and Co-N/HPC and the theoretical spectra simulated with the structures of a) Co–N_3C, b) Co–N_3, c) Co–N_2C_2-1, d) Co–N_4C_2-2, e) Co–N_1C_3, and f) Co–C_4 (color code: the brown, grey, and blue balls refer to C, N, and Co, respectively).

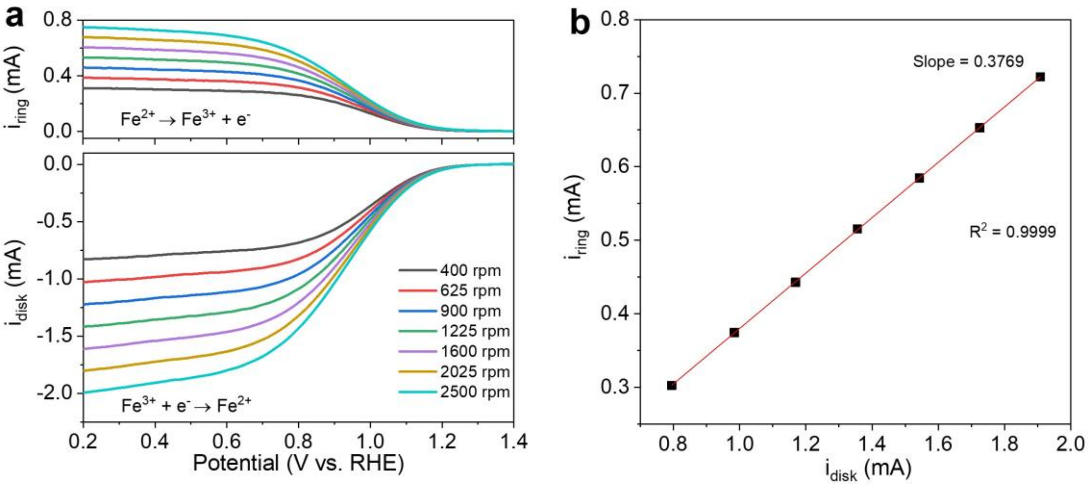

Figure S19. Collection efficiency calibration of the bare RRDE in 0.1 M KOH electrolyte with 10 mM $K_3Fe(CN)_6$: a) Linear sweep voltammetry curves recorded at various rotating speeds (Sweep rate: 20 mV s^{-1}; E_{ring} = 1.55 V vs. RHE). (b) Linear fitting of the diffusion-limited currents recorded on ring and disk electrodes.

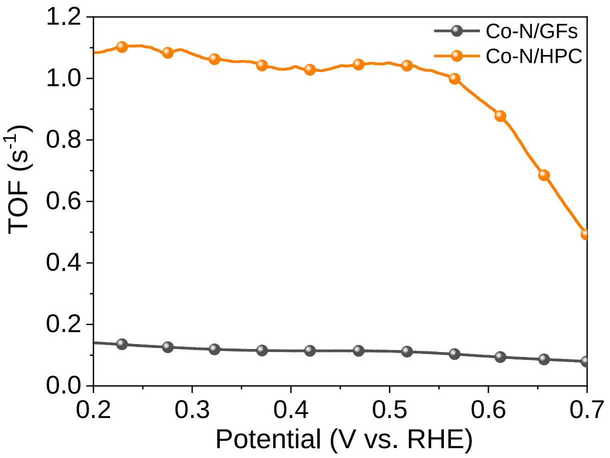

Figure S20. Calculated TOFs of Co-N/GFs and Co-N/HPC for the 2e$^-$-ORR as a function of applied potentials.

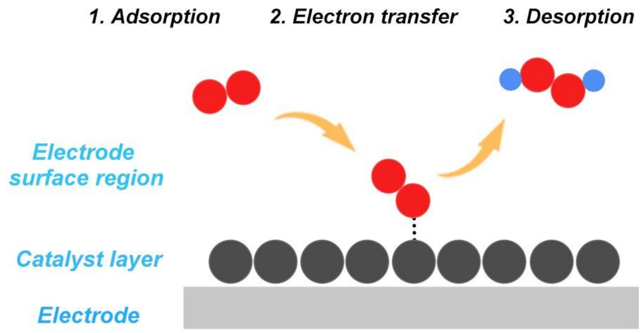

Figure S21. Schematic illustration of the electrochemical process of 2e$^-$-ORR on the electrode.

Generally, the ORR process on the disk electrode involves multiple elementary steps, including reactants/intermediates adsorption, chemical reactions coupled to electron transfer, and desorption (*Energy Environ*. *Sci*. **2022**, 15, 1172-1182). Proton-coupled electron transfer is usually kinetically unfavorable due to the energy barrier of O–O bond breakage. Correspondingly, the Tafel slope value of 129.4 mV dec^{-1} for metal-free HPC indicates electron transfer as the rate-determining step for the reduction of oxygen at high potentials. In contrast, the Tafel slope value of 72.0 mV dec^{-1} observed with the Co-N/GFs catalyst suggests that the ORR on that catalyst may be determined by the migration of adsorbed oxygen intermediates (*J*. *Am*. *Chem*. *Soc*. **2021**, 143, 7819). In the case of Co-N/HPC, the Tafel slope of 102.2 mV dec^{-1} is indicative of a more complicated ORR process, with the rate-determining step likely involving both the migration of intermediates and electron transfer (*J*. *Electrochem*. *Soc*. **2012**, *159*, H864-H870). The lower Tafel slope value than that of the HPC suggests that the electron transfer step becomes faster. Meanwhile, the difference in Tafel slope values for Co-N/GFs and Co-N/HPC also implies the different nature of the active sites toward the ORR.

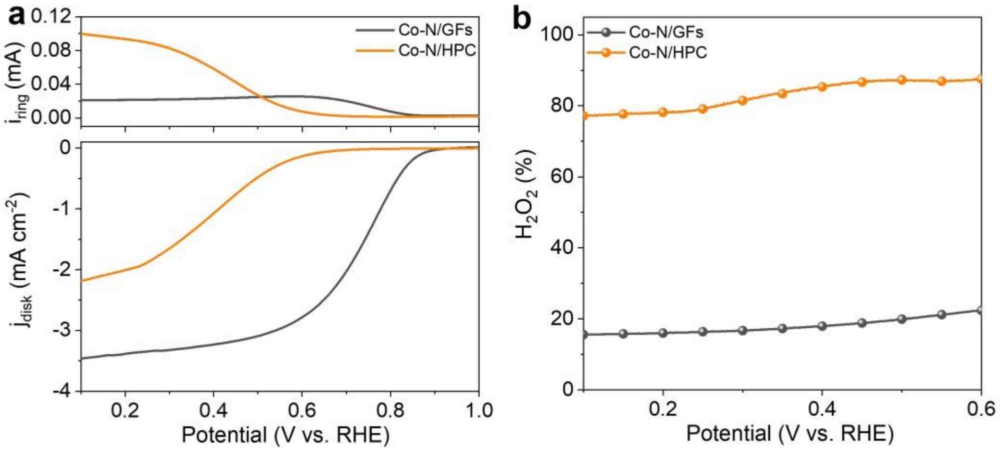

Figure S22. a) ORR LSV curves of Co-N/HPC and Co-N/GFs measured in O_2-saturated 0.1 M $HClO_4$ at a rotation speed of 1600 rpm. b) The corresponding H_2O_2 selectivity as determined by RRDE.

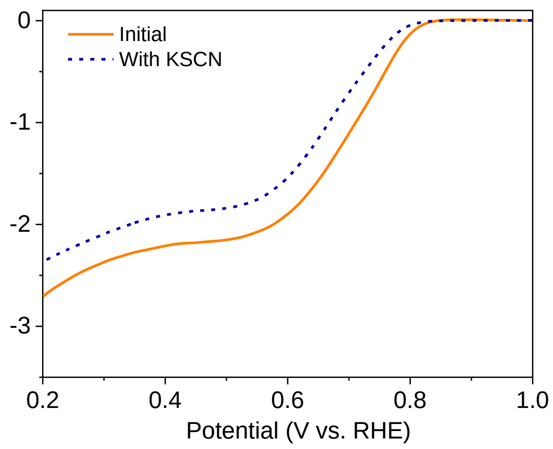

Figure S23. ORR LSV curves of Co-N/HPC in O_2-saturated 0.1 M KOH with and without KSCN.

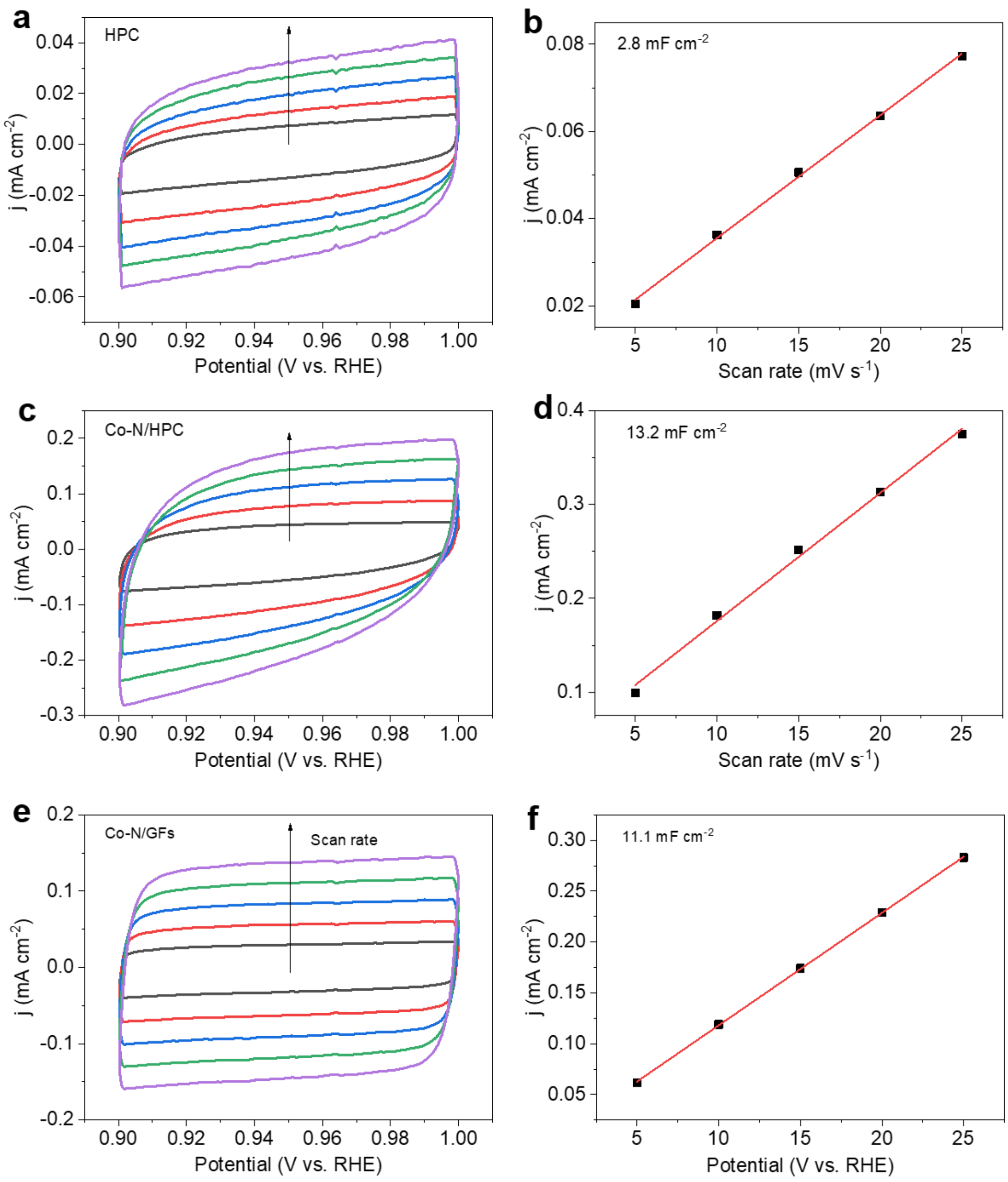

Figure S24. CV curves of a) HPC, c) Co-N/HPC, and e) Co-N/GFs obtained in the capacitance region at scan rates of 5, 10, 15, 20, and 25 mV s^{-1}. b, d, f) Corresponding electrochemical double-layer capacitances.

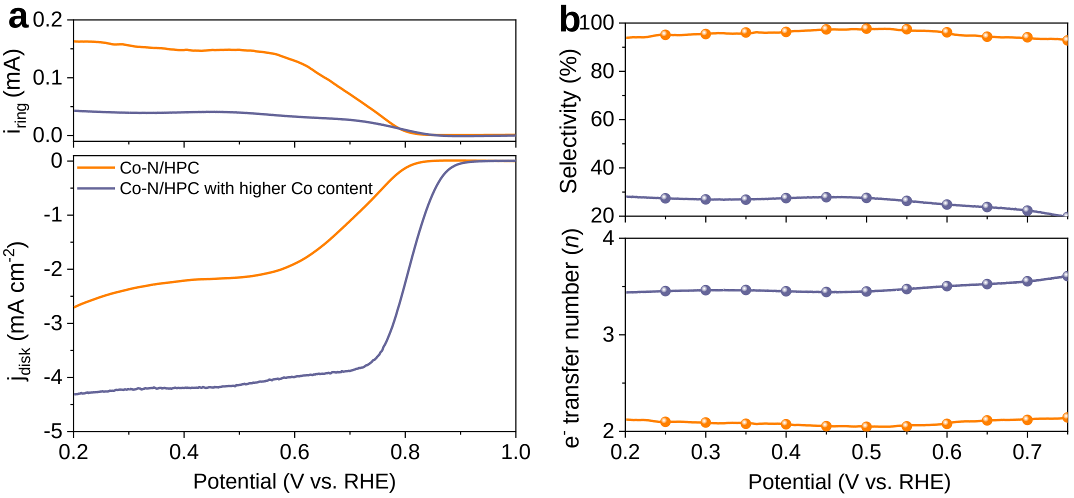

Figure S25. a) ORR LSV curves of Co-N/HPC catalysts with different Co contents in O_2-saturated 0.1 M KOH. b) Calculated 2e$^-$ selectivity and electron transfer number (n) as a function of applied potentials.

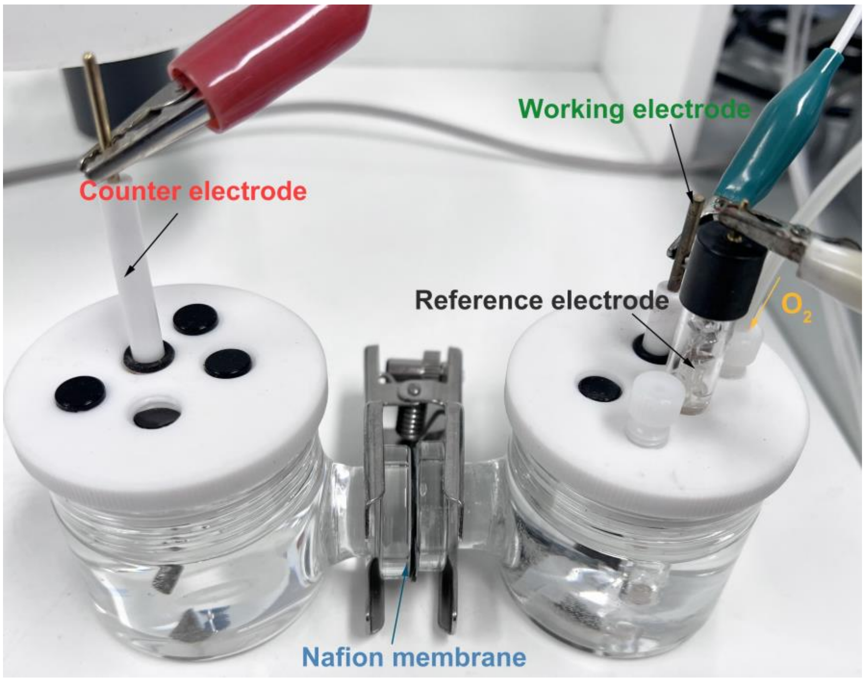

Figure S26. Digital photograph of the H-cell setup for electrochemical synthesis of H_2O_2.

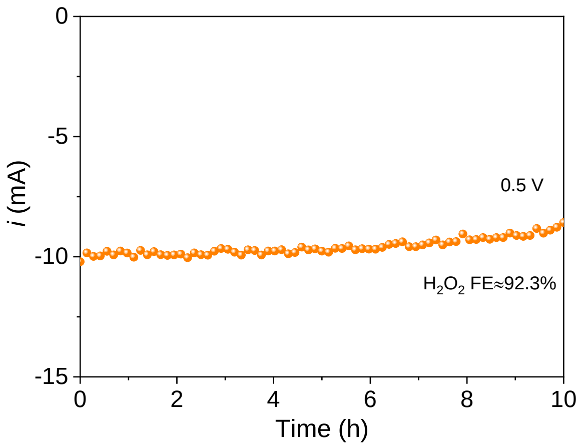

Figure S27. Stability test of Co-N/HPC coated carbon paper electrode in O_2-saturated 0.1 M KOH in the H-type cell under a fixed potential of 0.50 V vs. RHE.

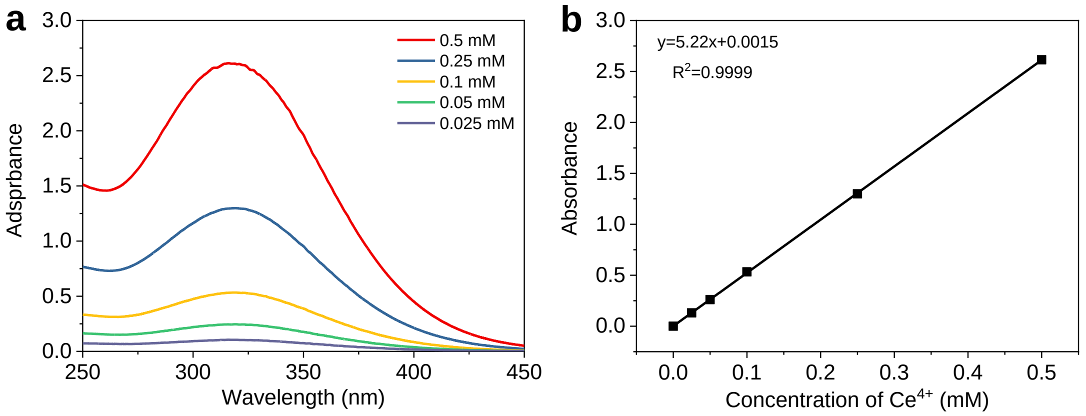

Figure S28. a) UV-vis spectra of Ce^{4+} ions with different concentrations and b) corresponding linear fitting of absorbance at 319 nm wavelength.

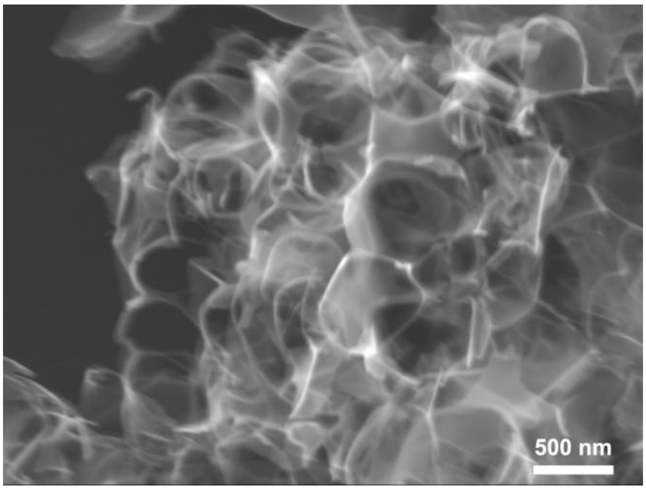

Figure S29. SEM image of Co-N/OHPC.

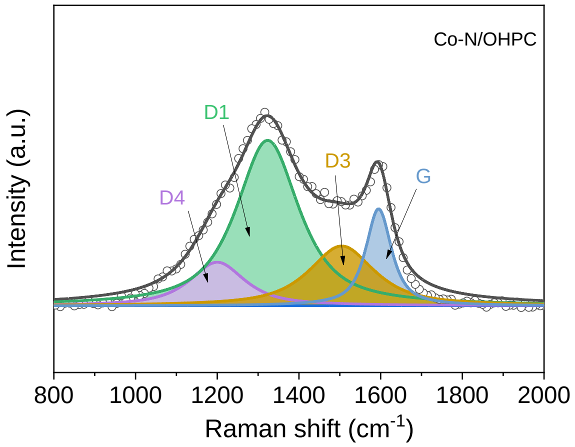

Figure S30. Raman spectrum of Co-N/OHPC.

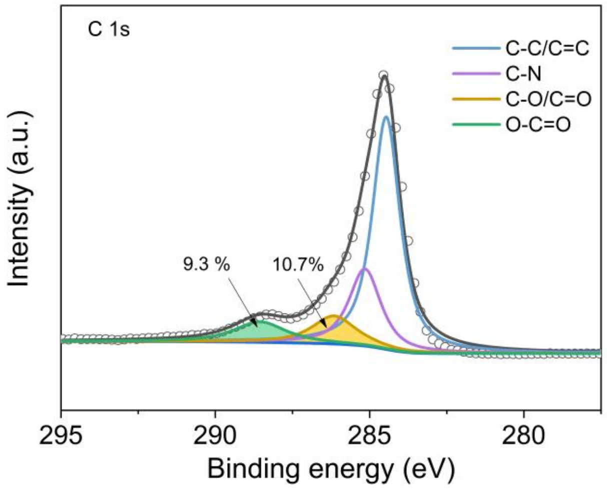

Figure S31. High-resolution C 1s spectrum of Co-N/OHPC.

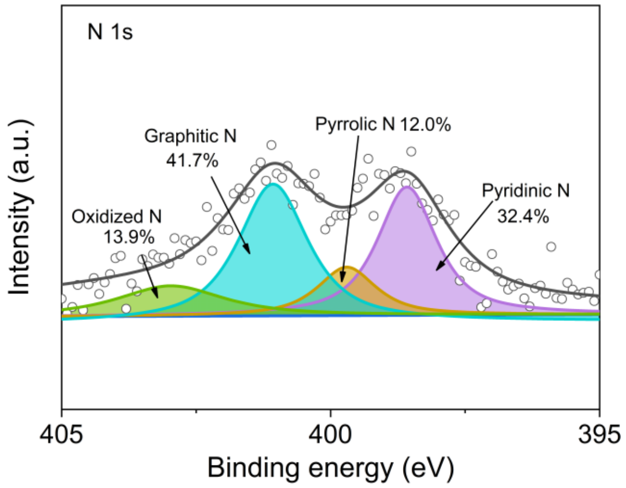

Figure S32. High-resolution N 1s spectrum of Co-N/OHPC.

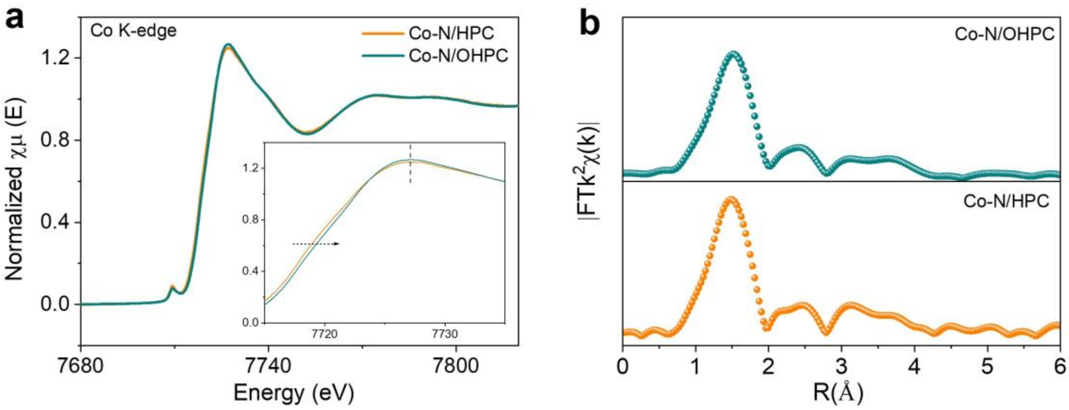

Figure S33. a) Co K-edge XANES and b) FT-EXAFS spectra of Co-N/HPC and Co-N/OHPC.

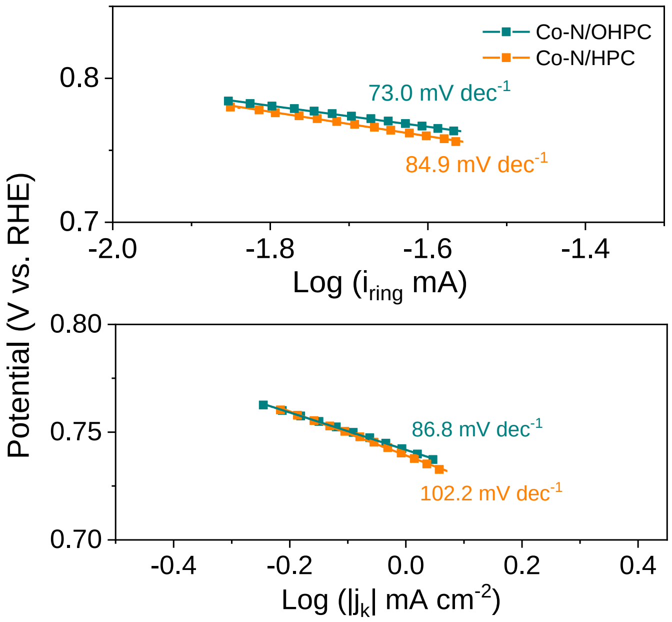

Figure S34. Tafel plots of Co-N/HPC and Co-N/OHPC.

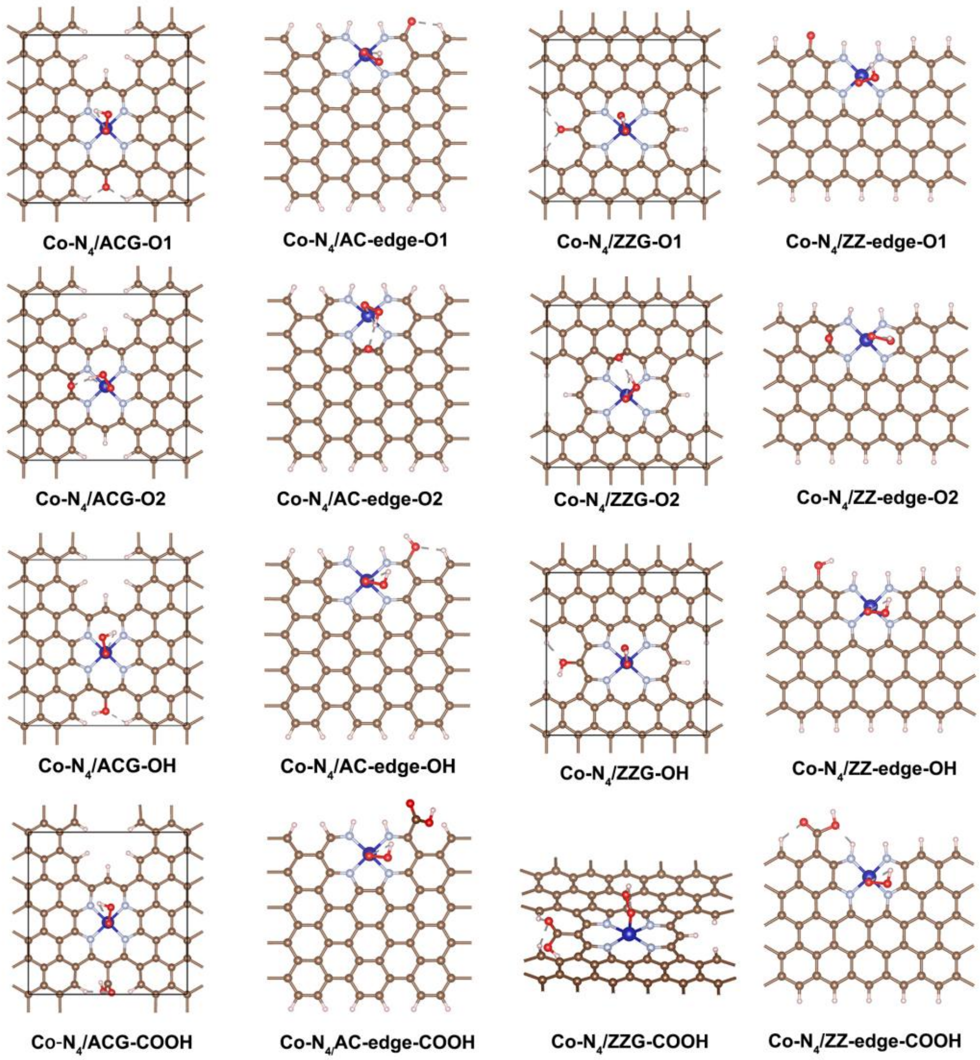

Figure S35. Optimized structural models of different Co–N_4 configurations with the modification of oxygen functional groups, including –OH, C–O, C=O, and –COOH (color code: the brown, grey, red, blue, and pink balls refer to C, N, O, Co, and H atoms, respectively).

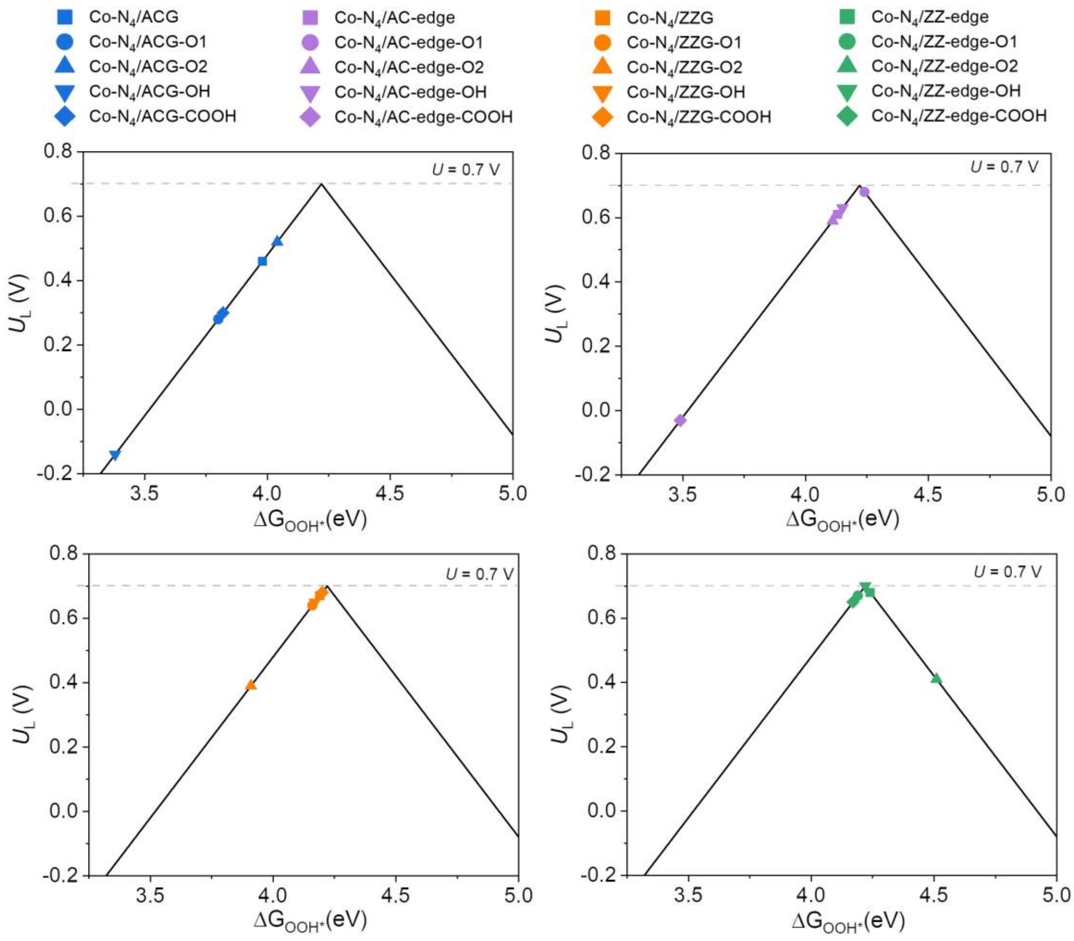

Figure S36. Calculated volcano plots of oxygen functional groups modified Co–N_4 configurations for $2e^-$-ORR (the U_L is plotted as a function of ΔG_{OOH^*}).

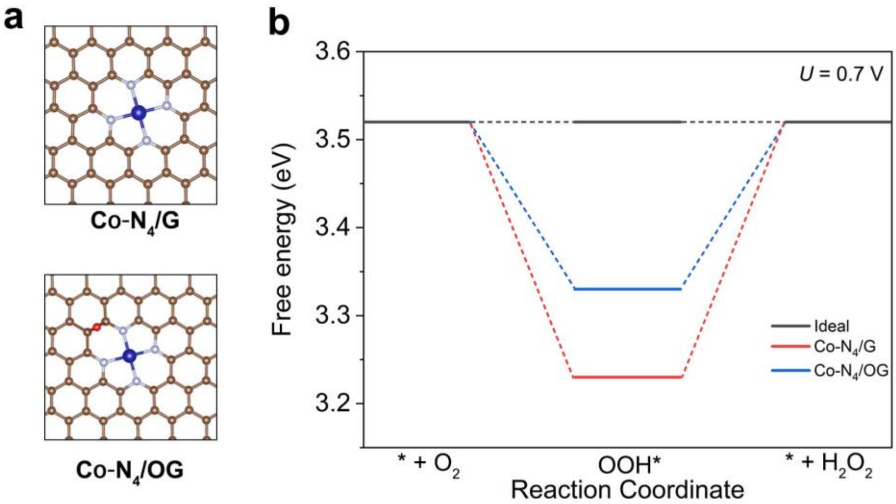

Figure S37. a) Optimized structural models of Co–N_4/G and Co–N_4/OG with the epoxy group (color code: the brown, grey, red, blue, and pink balls refer to C, N, O, Co, and H atoms, respectively). b) Free energy diagrams of 2e$^-$-ORR at U = 0.7 V.

Table S1. Summary of computed Bader charges, ΔG_{OOH^*}, and U_L of different Co–N_4 configurations.

Model	Bader charge (|e|)	ΔG_{OOH^*} (eV)	U_L (V)
Co-N_4/G	0.87	3.93	0.41
Co-N_4/ACG	0.91	3.98	0.46
Co-N_4/AC-edge	0.91	4.13	0.61
Co-N_4/ZZG	0.97	4.19	0.67
Co-N_4/ZZ-edge	0.98	4.24	0.68

Table S2. Fitting parameters of Co K-edge FT-EXAFS.

Sample	Scattering pair	CN	R(Å)	σ(Å^2)	ΔE_0 (eV)	R factor
Co-N/HPC	Co-N	3.9	1.98	0.0015	2.2	0.0127
Co-N/GFs	Co-N	4.3	1.89	0.0013	0.5	0.0201

CN is the coordination number; R is interatomic distance; ΔE_0 is the inner potential correction; σ is the Debye-Waller factor; R factor indicates the goodness of the fitting. The larger R value of Co-N/HPC than Co-N/GFs suggests the existence of a longer Co−N bond, which may be caused by the tensile strain induced by armchair edges.[7-8]

Table S3. Comparison of 2e⁻-ORR performances between Co-N/HPC and other reported electrocatalysts in alkaline media.

Catalysts	Selectivity @ (Potential V vs. RHE)	Mass activity (A g_{cat}^{-1}@V)	Production capacity	Faradaic efficiency (%)	ref
Co-N/HPC	98%@0.52	41.8@0.65	~1.72 mol h^{-1} g_{cat}^{-1}	92.3	This work
Co-N/HPC after durability	95.6@0.52	67.70@0.65	-	-	
Co_1-NG(O)	~82%@0.1	155@0.65	418±19 mmol h^{-1} g_{cat}^{-1}	-	[9]
Co-N_2-C/HO	91.3@0.7	44.4@0.65	6912 mmol h^{-1} g_{cat}^{-1}	-	[10]
Co-POC-O	82%@0.7	-	813 mg L^{-1} h^{-1}	64.1	[11]
Mo_1/OSG-H	~95% at 0.5	-	-	-	[12]
Ni-N_2O_2/C	96%@0.4	-	5.90 mol h^{-1} g_{cat}^{-1}	91	[13]
NiN_x/C-$AQNH_2$	80-85%@0.2-0.8	-	-	-	[14]
Fe-CNT	> 95%@0.7	-	~1.6 mol h^{-1} g_{cat}^{-1}	95.4	[15]
W_1/NO-C	90-98%@0.2-0.8	-	~1.2 mol h^{-1} g_{cat}^{-1}	95	[16]
O–C(Al)	97%@0.7	23.9@0.7		-	[17]
Ni MOF NSs	~98	-	~80 mmol h^{-1} g_{cat}^{-1}	96%	[18]
O-CNT	~90% at 0.45	-	~111.7 mmol h^{-1} g_{cat}^{-1}	-	[19]
GOMC	> 90%	~12.1@0.75	820 ± 70 mg L^{-1}	~99	[20]

N-FLG	~95% at 0.5	-	9.66 mol h^{-1} g_{cat}^{-1}	~100	[21]
B-C	~90% at 0.65	-	7.36 mmol h^{-1} cm^{-2}	~85	[22]
HCNFs	~97% at 0.60	220@0.65	6.37 mol h^{-1} g_{cat}^{-1}	-	[23]

References

[1] Z. Su, H. Y. Ling, M. Li, S. Qian, H. Chen, C. Lai, S. Zhang, *Carbon Energy* **2020**, *2*, 265-275.

[2] a) G. Kresse, J. Furthmüller, *Comput. Mater. Sci.* **1996**, *6,* 15. b) G. Kresse, D. Joubert, *Phys. Rev. B* **1999**, *59*, 1758.

[3] J. P. Perdew, K. Burke, M. Ernzerhof, *Phys. Rev. Lett.* **1996**, *77,* 3865.

[4] a) L. Zhong, S. Li, *ACS Catal*. 2020, 10, 4313-4318; b) X. Yang, D. Xia, Y. Kang, H. Du, F. Kang, L. Gan, J. Li, *Adv. Sci*. **2020**, *7,* 2000176.

[5] S. Grimme, *J. Comput. Chem.* **2006**, *27,* 1787-1799.

[6] a) L. Zhang, R. Si, H. Liu, N. Chen, Q. Wang, K. Adair, Z. Wang, J. Chen, Z. Song, J. Li, M. N. Banis, R. Li, T. K. Sham, M. Gu, L. M. Liu, G. A. Botton, X. Sun, *Nat. Commun*. **2019**, *10*, 4936; b) A. Kumar, V. Q. Bui, J. Lee, L. Wang, A. R. Jadhav, X. Liu, X. Shao, Y. Liu, J. Yu, Y. Hwang, H. T. D. Bui, S. Ajmal, M. G. Kim, S. G. Kim, G. S. Park, Y. Kawazoe, H. Lee, *Nat. Commun*. **2021**, *12*, 6766.

[7] G. Chen, Y. An, S. Liu, F. Sun, H. Qi, H. Wu, Y. He, P. Liu, R. Shi, J. Zhang, A. B. Kuc, U. Kaiser, T. Zhang, T. Heine, G. Wu, X. Feng, *Energy Environ. Sci*. **2022**, *15*, 2619-2628.

[8] Y. H. Jeong, S. C. Kim, S. R. E. Yang, *Phys*. Rev. *B* 2015, 91, 205441.

[9] E. Jung, H. Shin, B. H. Lee, V. Efremov, S. Lee, H. S. Lee, J. Kim, W. Hooch Antink, S. Park, K. S. Lee, S. P. Cho, J. S. Yoo, Y. E. Sung, T. Hyeon, *Nat. Mater*. **2020**, *19*, 436

[10] H. Gong, Z. Wei, Z. Gong, J. Liu, G. Ye, M. Yan, J. Dong, C. Allen, J. Liu, K. Huang, R. Liu, G. He, S. Zhao, H. Fei, *Adv. Funct. Mater*. **2021**, *32*, 2106886

[11] B. Q. Li, C. X. Zhao, J. N. Liu, Q. Zhang, *Adv. Mater*. **2019**, 31, 1808173.

[12] C. Tang, Y. Jiao, B. Shi, J. N. Liu, Z. Xie, X. Chen, Q. Zhang, S. Qiao, *Angew. Chem. Int. Ed*. **2020**, *59*, 9171-9176.

[13] Y. Wang, R. Shi, L. Shang, G. I. N. Waterhouse, J. Zhao, Q. Zhang, L. Gu, T. Zhang, *Angew. Chem. Int. Ed*. **2020**, *59*, 13057-13062.

[14] X. Li, S. Tang, S. Dou, H. J. Fan, T. S. Choksi, X. Wang, *Adv. Mater*. **2021**, *34*, 2104891.

[15] K. Jiang, S. Back, A. J. Akey, C. Xia, Y. Hu, W. Liang, D. Schaak, E. Stavitski, J. K. Norskov, S. Siahrostami, H. Wang, *Nat. Commun*. **2019**, *10*, 3997.

[16] F. Zhang, Y. Zhu, C. Tang, Y. Chen, B. Qian, Z. Hu, Y. C. Chang, C. W. Pao, Q. Lin, S. A. Kazemi, Y. Wang, L. Zhang, X. Zhang, H. Wang, *Adv. Funct. Mater.* **2021**, *32*, 2110224.

[17] Q. Yang, W. Xu, S. Gong, G. Zheng, Z. Tian, Y. Wen, L. Peng, L. Zhang, Z. Lu, L. Chen, *Nat. Commun.* **2020**, *11*, 5478.

[18] M. Wang, X. Dong, Z. Meng, Z. Hu, Y. G. Lin, C. K. Peng, H. Wang, C. W. Pao, S. Ding, Y. Li, Q. Shao, X. Huang, *Angew. Chem. Int. Ed.* **2021**, *60*, 11190-11195.

[19] Z. Lu, G. Chen, S. Siahrostami, Z. Chen, K. Liu, J. Xie, L. Liao, T. Wu, D. Lin, Y. Liu, T. F. Jaramillo, J. K. Nørskov, Y. Cui, *Nat. Catal.* **2018**, *1*, 156-162.

[20] Y. J. Sa, J. H. Kim, S. H. Joo, *Angew. Chem. Int. Ed.* **2019**, *58*, 1100-1105.

[21] L. Li, C. Tang, Y. Zheng, B. Xia, X. Zhou, H. Xu, S. Z. Qiao, *Adv. Energy Mater.* **2020**, *10*, 2000789.

[22] Y. Xia, X. Zhao, C. Xia, Z. Y. Wu, P. Zhu, J. Y. T. Kim, X. Bai, G. Gao, Y. Hu, J. Zhong, Y. Liu, H. Wang, *Nat. Commun.* **2021**, *12*, 4225.

[23] K. Dong, J. Liang, Y. Wang, Z. Xu, Q. Liu, Y. Luo, T. Li, L. Li, X. Shi, A. M. Asiri, Q. Li, D. Ma, X. Sun, *Angew. Chem. Int. Ed.* **2021**, *60*, 10583-10587.
